# Efficacy and safety of human umbilical cord-derived mesenchymal stem cells for COVID-19 pneumonia: a meta-analysis of randomized controlled trials

**DOI:** 10.1186/s13287-023-03286-8

**Published:** 2023-05-04

**Authors:** Qinxue Liu, Fengjie Ma, Yizhi Zhong, Gaojian Wang, Li Hu, Yaping Zhang, Junran Xie

**Affiliations:** 1 Department of Anesthesiology, Sir Run Run Shaw Hospital, School of Medicine, Zhejiang University, No.3 East Qingchun Road, Jianggan District, Hangzhou, 310016 China; 2 Department of Anesthesiology, Second Affiliated Hospital of Jiaxing University, No.1518 North Huancheng Road, Nanhu District, Jiaxing, 314000 China

**Keywords:** Coronavirus disease-19 (COVID-19), Mesenchymal stem/stromal cells (MSCs), Cytokines, Efficacy, Safety, Meta-analysis

## Abstract

**Background:**

Elevated levels of inflammatory factors are associated with poor prognosis in coronavirus disease-19 (COVID-19). However, mesenchymal stem cells (MSCs) have immunomodulatory functions. Accordingly, this meta-analysis aimed to determine the efficacy and safety of MSC-based therapy in patients with COVID-19 pneumonia.

**Methods:**

Online global databases were used to find relevant studies. Two independent researchers then selected and evaluated the studies for suitability while the Cochrane risk of bias tool determined the quality of all articles and Cochran's Q test and I^2^ index assessed the degree of heterogeneity in the principal studies. Statistical analysis was performed using Review Manager software, and the effect of each study on the overall estimate was evaluated by sensitivity analysis.

**Results:**

Seven studies were included in the meta-analysis, and all MSCs used in the trials were acquired from the umbilical cord. The results of these studies (n = 328) indicated that patients with COVID-19 pneumonia who received MSCs had a 0.58 risk of death compared with controls (95% CI = 0.38, 0.87; P = 0.53; I^2^ = 0%). In terms of inflammatory biomarkers, MSCs reduced the levels of C-reactive protein (n = 88; MD =  − 32.49; 95% CI =  − 48.43, − 16.56; P = 0.46; I^2^ = 0%) and interferon-gamma (n = 44; SMD =  − 1.23; 95% CI =  − 1.89, − 0.57; P = 0.37; I^2^ = 0%) in severe COVID-19 patients but had no significant effect on interleukin-6 (n = 185; MD =  − 0.75; 95% CI =  − 7.76, 6.27; P = 0.57; I^2^ = 0%). A summary of the data revealed no significant differences in adverse events (n = 287) or serious adverse events (n = 229) between the MSC and control groups.

**Conclusions:**

Infusion of umbilical cord-derived MSCs is an effective strategy for treating patients with COVID-19 pneumonia, with no noticeable adverse effects.

**Supplementary Information:**

The online version contains supplementary material available at 10.1186/s13287-023-03286-8.

## Introduction

Coronavirus disease 2019 (COVID-19), caused by severe acute respiratory syndrome coronavirus 2 (SARS-CoV-2), has spread rapidly and continuously worldwide since December 2019 [1]. The infectious sources of the novel coronavirus pneumonia are often unclear, the routes are diverse, and the clinical outcomes are highly variable [2]. The disease is self-limiting in most mild patients, but some severe patients experience tachypnea, decreased oxygen saturation, acute respiratory distress syndrome (ARDS), septic shock, multiple organ dysfunction syndrome, and even death [3]. A multicenter retrospective cohort study in Spain found a 30% mortality rate in severe COVID-19 patients admitted to the intensive care unit [4]. Therefore, COVID-19 has caused significant damage to human life and health. Some treatment measures, such as antiviral therapy, steroid therapy, respiratory support, and molecular therapy, play restricted roles in severe and critical COVID-19 patients. Thus, a safe and effective treatment method is urgently required.

SARS-CoV-2 enters the host cell when angiotensin-converting enzyme-2 (ACE2) receptors recognize and bind to its spike protein [5]. Another study showed that transmembrane serine protease 2 (TMPRSS2) is also involved in cell entry [6]. After entering the human body, the virus replicates and multiplies, causing the production and release of inflammatory factors that attempt to kill the virus [7]. However, the immune system response often gets out of control, which results in severe cytokine release syndrome (CRS) and ultimately causes multiple organ system failure (MOSF) and fatal respiratory distress [8]. In the first clinical data on SARS-CoV-2 infections, Huang et al. reported that critical patients had high levels of interleukin 2 (IL-2), IL-7, IL-10, GSCF, IP10, MCP1, MIPA, and TNF in their blood [9]. This flood of inflammatory cytokines is also called CRS. Subsequently, Lucas et al. found that early increases in cytokine levels were associated with worse outcomes for COVID-19, which indicates that the cytokine storm is crucial to the COVID-19-related deterioration and offers potential therapeutic targets [10]. Consequently, the timely treatment of the cytokine storm is essential. Various cytokines are involved in the cytokine storm. IL-6 and its downstream acute phase protein C-reactive protein (CRP) can be critical proinflammatory cytokines in cascade reactions and cytokine storms. For instance, Gordon et al. found that the administration of IL-6 receptor antagonist to severely ill COVID-19 patients receiving organ support in the intensive care unit improved their prognosis [11]. Likewise, interferon-gamma (IFN-γ) can lead to cell death in various cell types, damage vital organs, and is also a central effector cytokine in cellular immunity. For example, Karki et al. discovered that inhibition of TNF-α and IFN-γ reduced mortality in a model of the disease associated with cytokine storm [12]. Therefore, it is reasonable to use IL-6, CRP, and IFN-γ as biomarkers for monitoring the treatment of CRS.

Mesenchymal stem cells (MSCs) are pluripotent tissue stem cells originating from the early mesoderm. Due to their regenerative, angiogenic, and antifibrotic functions, and particularly their anti-inflammatory and immunomodulatory activity, they have recently attracted considerable interest in cell therapy [13]. MSCs can modulate innate and adaptive immune cells through direct contact or paracrine signals to decrease the levels of proinflammatory factors and increase those of anti-inflammatory factors [14]. As a result, they can inhibit CRS and repair the damage caused by the overactive immune response [15]. In addition, MSCs are negative for ACE2 receptor and TMPRSS2, indicating that MSCs are not at risk of being infected by SARS-CoV-2 [16]. MSCs also have considerable advantages for clinical use. For example, they can be isolated from various sources, readily harvested and stored, produced on a large scale, and differentiated into multiple cell types. They also exhibit invasiveness, chemotaxis in damaged tissues, and low immunogenicity and have no toxic adverse effects or ethical problems [17]. Hence, MSC-based immunomodulatory therapy is considered feasible for treating COVID-19.

Over 200 clinical studies of the MSC treatment of COVID-19 have already been registered. More than 20 studies have been published, including 10 randomized controlled trials (RCTs) [18–27]. Leng et al. recruited seven COVID-19 patients, from mild to severe and to critically ill, in early 2020 [28]. These patients received a single intravenous injection of 1 × 10^6^ cells/kg of ACE2-negative MSCs. The cells significantly improved outcomes without observed adverse effects. At the same time, Feng et al. conducted a pilot trial with 16 severe and critically ill COVID-19 patients [29]. Their experimental team increased the number of transplants to 4, with 1 day in between. The number of transplanted cells was 1 × 10^8^ each time. No infusion-related or allergic reactions occurred, and the oxygenation index and radiological performance improved. Since then, other authors have reported similar results regarding MSC safety and feasibility [26, 30–34]. However, trials with MSCs as interventions are currently in phase I or II and the number of COVID-19 patients who have received MSC therapy is small. In addition, the research settings and outcomes are inconsistent across the clinical studies. An urgent question is whether to conduct more extensive clinical trials and apply vigorous MSC therapies to cure COVID-19.

Meta-analyses can help clinicians, scientists, and policymakers to get up-to-date, high-quality information on specific topics by reducing the impact of randomness and obtaining complete data [35]. In addition, although some meta-analyses have examined the use of stem cells in the treatment of COVID-19 or ARDS, few of the analyses focused specifically on RCTs. In the present meta-analysis, we not only analyzed the changes in inflammatory factors in all kinds of COVID-19 patients, but also additionally excluded mild patients and compared the inflammatory factors only in severe COVID-19 patients [36–38]. Thus, our aim was to meta-analyze RCTs that used MSCs to treat COVID-19 since its first occurrence in 2019 in order to assess their efficacy and safety and analyze the potential challenges associated with such a treatment.

## Methods

### Search strategy

Until March 22, 2022, the PubMed, EMBASE, Cochrane Library, Web of Science, and ClinicalTrials databases were thoroughly searched. Using subject words and free words based on MSCs, we sought COVID-19 pneumonia treatments. Manual searches were conducted in addition to computerized searches. All search phrases are available in the appendix: Additional file 1: Table S1, Additional file 2: S2, Additional file 3: S3 and Additional file 4: S4.

### Eligibility criteria

We included RCTs evaluating the safety and effectiveness of MSCs as a therapeutic intervention in patients with confirmed COVID-19 pneumonia. The research included individuals with confirmed COVID-19 pneumonia (e.g., quantitative RT-PCR, antigen assay) [39]. We excluded the MSC secretome and restricted the intervention methods to MSCs from any known acceptable tissue source (e.g., bone marrow, adipose tissue, umbilical cord, dental pulp, placenta). Patients received standard treatment with or without placebo in the control group. All delivery routes were permissible (e.g., intravenous, aerosol inhalation, intramuscular) [40]. When the same research team followed the same group of patients in the short- and long-term, we selected complete papers with a follow-up time of less than 3 months and similar outcome indicators. Excluded were clinical trials involving pregnant or breastfeeding patients or those with other immunocompromised states. We also excluded literature that did not report original data or did not make original data available and any articles published in languages other than English.

### Data extraction

Two researchers independently examined the literature, collected data, and compared their results. Any conflicts that occurred were addressed and resolved with the aid of a third investigator. The original author was contacted to provide any missing information. Forms for the routine extraction of data were devised and tested and included the following: (1) basic information on the included research, such as author, publication year, and country; (2) essential characteristics of research subjects, including sample size, follow-up time, age, male-to-female ratio, comorbidities, and baseline treatment; (3) essential intervention features, such as tissue source of MSCs, dose, route of administration, frequency, and adherence to minimal requirements from the International Society for Cell & Gene Therapy (ISCT) [41]; and (4) all pertinent data on primary and secondary outcomes. We used WebPlotDigitizer (version 4.5, https://automeris.io/WebPlotDigitizer/) to digitize graphs and extract data from publications that did not present data directly.

### Quality assessment

We used the Cochrane risk of bias assessment tool [42] built into Review Manager software (RevMan, Cochrane, version 5.4, https://training.cochrane.org/online-learning/core-software/revman/revman-5-download) to assess the quality of the RCTs as follows: (1) whether the allocation sequence was random; (2) whether the allocation was concealed; (3) whether blinding was used; (4) the integrity of the outcome data; (5) whether outcomes were selectively reported; and (6) whether there was any additional bias. Two independent reviewers separately evaluated the possibility of discrimination, and the findings were cross-checked. A third author handled any conflicts that arose after publication.

### Study selection

EndNote loaded the search results before filtering them. After deleting duplicates, two independent reviewers independently evaluated the study title and abstract. After the identification of potentially relevant titles and abstracts, the full text was independently assessed to ensure eligibility. In the event of a disagreement between the two reviewers, a third senior member of the team was contacted to reach a decision.

### Statistical analysis

The meta-analysis used RevMan. The risk ratio (RR) between the control and experimental groups was determined for dichotomous outcomes. The mean difference (MD) was calculated for continuous results. When the measuring techniques and research units were diverse, or the variation in means between different studies was too significant, the standardized mean difference (SMD) was used as the effect size. These data are reported with their 95% confidence interval (95% CI). The included studies reported the median, maximum, and minimum values, which translated to the mean and standard deviation and were then analyzed collectively. We used the I^2^ index and the Cochran Q test to evaluate study heterogeneity. We operated the fixed-effects model for meta-analysis when P ≥ 0.1 in the Q statistic or I^2^ < 50%. If P < 0.1 or I^2^ ≥ 50%, there was at least considerable statistical heterogeneity, indicating the need for additional study. After the removal of significant clinical and methodological heterogeneity, a random-effects model was applied in the meta-analysis. After removing individual studies, we conducted a sensitivity analysis and repeated the meta-analysis to assess the influence of each RCT on the overall estimate. A meta-analysis was performed only when at least three RCTs reported the same result. P < 0.05 was considered significant for all analyses.

## Results

### Search results

**Fig. 1:**
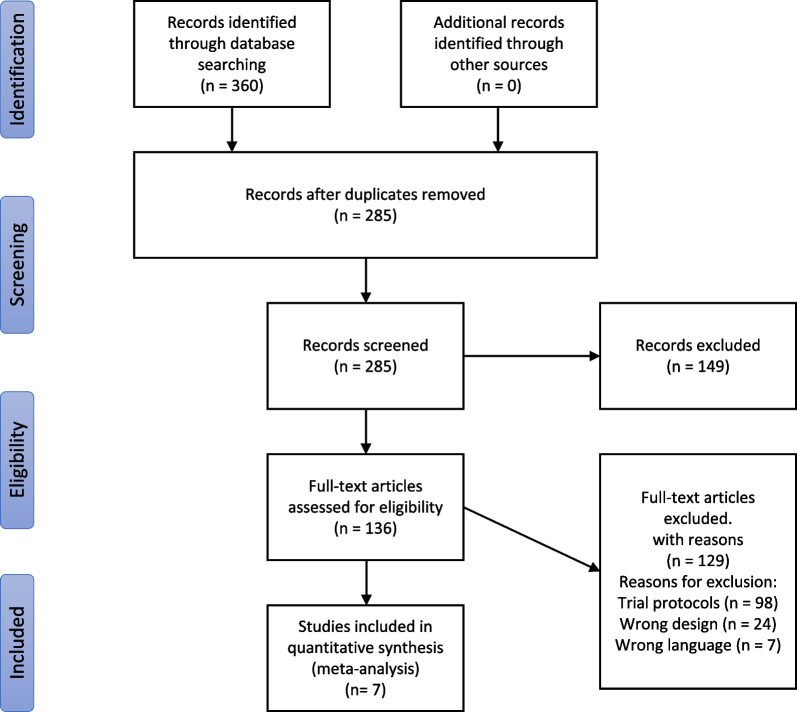
Flowchart of the search strategy and study selection

A flowchart of the literature search and screening approach is shown in Fig. 1. After the removal of duplicates, our literature search yielded 360 unique records. We excluded articles for various reasons, including trial protocol only (n = 98), uncontrolled case series (n = 13), non-RCT (n = 4), only MSC secretome cells (n = 3), wrong population (n = 1), other reasons (n = 3), and non-English languages (n = 7; two Italian, two Chinese, and three Russian). Seven articles met the criteria for inclusion in our analysis and were finally included in this meta-analysis [18, 19, 22–24, 26, 27].

### Quality assessment of the included studies

**Fig. 2:**
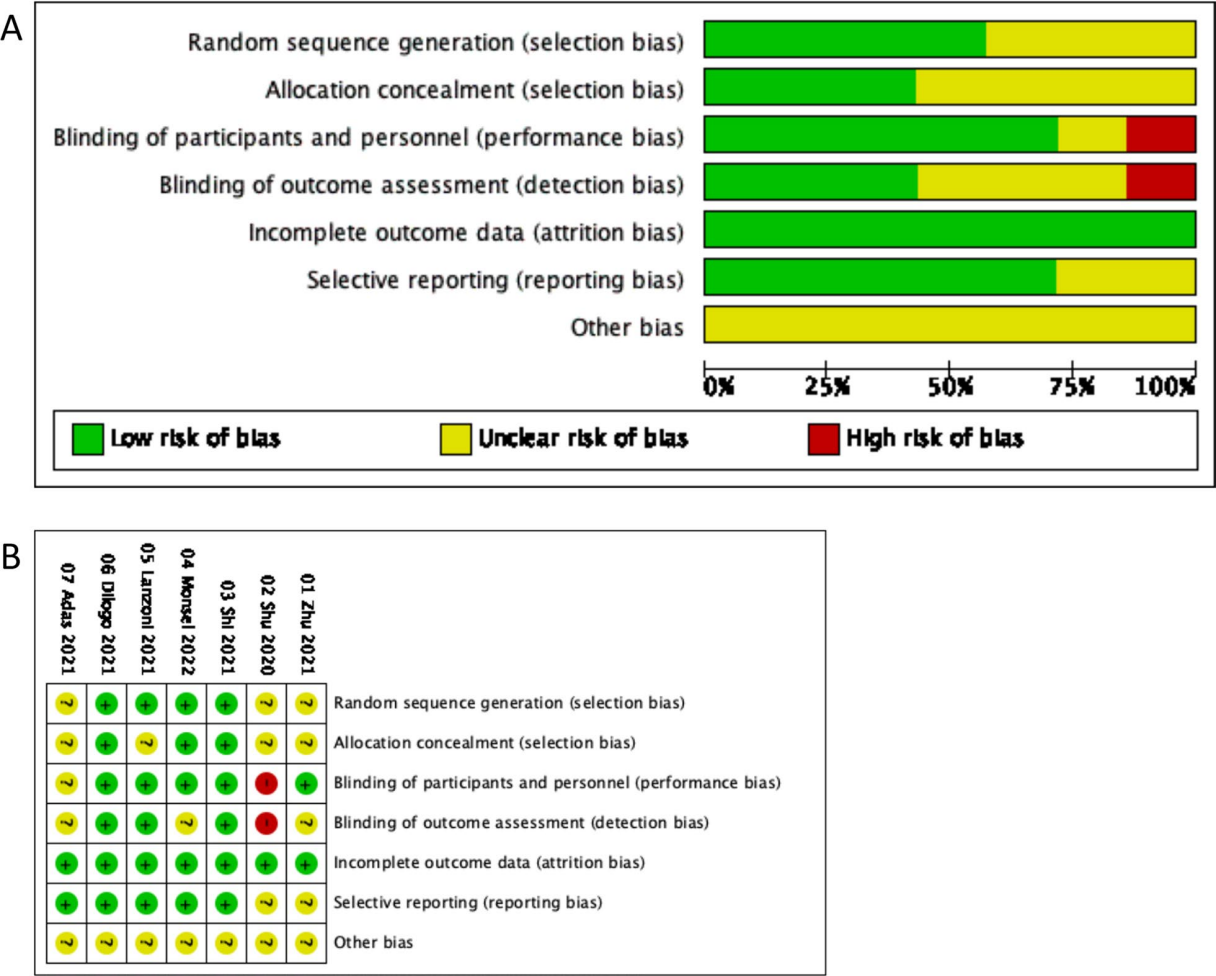
Risk of bias assessment. **A** Graph of the risk of bias for the included studies. **B** Graph of the risk of bias overview for the included studies

Figure 2 shows the results of the quality assessment. The risk of bias assessment was performed on the seven included studies.

Random sequence generation: four studies used a computerized random number method for randomization and were rated as “unclear risk.” The remaining three studies were only described as random groupings, and the technique is unknown, so they were given an “unclear risk” rating.

Allocation concealment: three articles mentioned the use of computers to assign third-party central allocation and were rated as “unclear risk.” The other four articles did not say if allocation concealment was used or not, so they were given an “unclear risk” rating.

Blinding: five studies blinded participants and investigators and were rated as “low risk”. One article was an open-label trial and was rated as “high risk”. One article did not discuss blinding and was rated as “unclear risk”.

Incomplete outcome data: the seven articles stated that there was no case culling or drop outs, that the numbers and reasons for drop outs were the same between groups, or that 20% of the data were missing, but that was not enough to change the size of the effect, or that an appropriate method was used to handle missing values and were deemed “low risk”.

Selective reporting: five studies fully reported the outcome indicators and were judged to be “low risk”. The registration information of the two studies did not specify the specific detection indicators and methods in detail, and they were regarded as “unclear risk”.

Other bias: There are insufficient research results to know if there were other biases.

### Study characteristics

Table 1 summarizes the features of the studies. Four of the seven RCTs were phase I/II or phase II clinical studies conducted in five countries: three in China and one each in France, the USA, Indonesia, and Turkey. Included were 328 individuals with proven COVID-19 pneumonia; 169 patients were treated with MSCs and 159 patients served as controls. All seven trials (n = 328) reported mortality results. Five studies (n = 243) compared inflammatory markers, including CRP (n = 103), IFN-γ (n = 144), and IL-6 (n = 185). Six studies measured adverse events (AEs) (n = 287), and five studies estimated severe AEs (SAEs) (n = 229).

**Table 1:** Characteristics of included studies

	Author	Country	Study type	Patient number			Mean age			Female sex (%)			F/U	Outcomes
				Total	MSC	Ctrl	Total	MSC	Ctrl	Total	MSC	Ctrl		
2021	Zhu et al.	China	phase II RCT	58	29	29	63.49	62.04	64.93	62.1	58.60	65.5	4W	mortality, AE, CRP
2020	Shu et al.	China	RCT	41	12	29	58.78	61	57.86	41.46	33.33	44.83	4W	mortality, CRP, IL-6
2021	Shi et al.	China	phase 2 RCT	100	65	35	60.45	60.72	59.94	44	43.08	45.71	4W	mortality, AE, SAE, IL-6, IFN-γ
2022	Monsel et al.	France	phase 2b RCT	45	21	24	63.57	64.00	63.20	17.78	19.05	16.67	4W	mortality, AE, SAE
2021	Lanzoni et al.	America	phase 1/2a RCT	24	12	12	59	58.58	58.83	45.8	58.30	33.3	1 M	mortality, AE, SAE, IL-6, IFN-γ
2021	Dilogo et al.	Indonesia	RCT	40	20	20	NR	NA	NA	25	25	25	1 M	mortality, AE, SAE
2021	Adas et al.	Turkey	RCT	20	10	10	NA	NR	NR	NA	NR	NR	3 M	mortality, AE, SAE, CRP, IL-6, IFN-γ

MSCs, mesenchymal stem/stromal cells Ctrl; RCT, Randomized controlled trial; AE, adverse event; SAE, Serious adverse event; CRP, C-reaction protein; IL-6, Interleukin-6; IFN-γ, Interferon-gamma; F/U, follow-up; W, week; M, month

### Patient characteristics

The characteristics of the trial patients are outlined in Table S5, and Table 2 shows the features of the patients in the trials. The average age of the 328 participants in the research was between 58 and 64 years old, and 38.41% were female. The average age of the 169 patients who underwent MSC-based therapy was between 59 and 64 years old and 65 of the patients, or 38.46%, were female. In the control group, the average age of the patients was between 58 and 64 years old, with 61 female patients constituting 38.36% of the total. The distribution of mild/moderate, severe, and critically ill COVID-19 patients receiving MSC-based treatment was comparable to that of the COVID-19 patients in the control group. However, there were fewer hypertensive patients in the intervention group than in the control group (27.81% vs. 35.22%). The control and intervention groups seemed to be evenly matched for various comorbidities, such as cardiovascular disease, chronic respiratory illness, diabetes, liver and renal disease, obesity, and smoking. Baseline treatments such as oxygen therapy, non-invasive ventilation, invasive ventilation, cortisol, antibiotics, and other drug use at enrollment showed no difference between the control and intervention groups.

**Table 2:** Comparison of MSC and control group patient characteristics

Patient characteristics	Total	MSC	Ctrl
Number of patients	328	169	159
Number of females	126	65	61
Mean age	61.25	61.33	61.1
*Covid-19 severity*			
Mild/moderate	31	15	16
Severe	231	121	110
Critical	66	33	33
*Comorbidities*			
Heart disease	30	18	12
Hypertension	103	47	56
Chronic respiratory disease	9	5	4
Smoker	2	0	2
Obesity	29	18	11
Diabetes	64	32	32
History of liver and kidney disease	12	4	8
Others^a^	10	6	4
Total	259	130	129
*Baseline therapy*			
Oxygen therapy	154	84	70
Non-invasive mechanical	30	18	12
Invasive mechanical	31	11	20
Corticosteroids	80	43	37
Antibiotic	76	45	31
Anti-virus therapy	98	54	44
Immunomodulatory drugs	10	4	6
On vasopressors	19	5	14
Total	498	264	234

Other^a^ include stroke, cerebrovascular disease, active neoplasia, immunodeficiency, cancer, tuberculosis

### Intervention characteristics

Table 3 summarizes the features of the intervention. All seven RCTs used allogeneic MSCs obtained from the umbilical cord. Two studies reported MSCs from two women, but the remaining five studies did not name the sources. According to ISCT criteria, five studies identified and reported the characterization of the MSCs, most of which were positive for CD73, CD90, and CD105 and negative for CD34, CD45, CD14, CD11b, CD79, CD19, and HLA-DR. However, only three of the seven studies examined whether the MSCs satisfied all three minimal ISCT requirements, with one fulfilling all three and two reaching one criterion. The number of passages at which the MSCs were taken varied across the studies, with five reports saying that the MSCs were harvested from 3 to 6 passages and two failing to disclose this information. MSC dosages varied based on about 60 kg of adult weight: three studies used MSCs at 1 × 10^6^/kg, two studies used MSCs at 2 × 10^6^/kg, and two studies used MSCs at 3 × 10^6^/kg. In three trials, 139 patients had one MSC infusion, 24 patients in one study received two, and 165 patients in three studies received three. In all seven studies, the MSCs were administered via intravenous injection. Five studies indicated that the control group patients received the same amount of vehicle and level of treatment as the intervention group patients. Only Adas (2021) followed up with the patient for three months after MSC treatment, whereas the other authors followed up for almost a month.

**Table 3:** Description of included clinical trial interventions

Year	Author	Source	Type	Donor	Surface markers	P	Cultural media	Dose per kg	Viability %	F	Route	Control	ISCT^*^
2021	Zhu et al.	UC	NR	NR	NR	NR	NR	1 × 10^6^	NR	1	ivdrip	100 mL 0.9% saline solution	NR
2020	Shu et al.	UC	allogenic	NR	Positive CD73, CD90, CD105 negative CD34, CD45, CD14 or CD11b, CD79α, CD19, HLA-DR	3–5	NR	2 × 10^6^	NR	1	ivdrip	NR	2
2021	Shi et al.	UC	allogenic	NR	Positive CD73, CD90, CD105 negative CD34, CD45, CD14 or CD11b, CD79α, CD19, HLA-DR	5	DMEM/F12 + 10%FBS	0.67 × 10^6^	NR	3	ivdrip	100 ml placebo	1,2,3
2022	Monsel et al.	UC	allogenic	2 (female)	Positive CD73, CD90, CD105 Negative CD45, CD34, CD11b, CD19, HLA-DR	4	Nutristem® MSC XF Basal Medium + Nutristem® MSC XF Supplement Mix + 5% irradiated platelet lysate MultiPL100i + sodium heparin 2 IU/mL	3 × 10^6^	78.4 ± 5.3	3	ivdrip	150 ml 0.5% albumin or placebo	2
2021	Lanzoni et al.	UC	allogenic	2 (female)	positive CD90, CD105 negative CD34/CD45 < 5%	NR	NR	2 × 10^6^	96.2 ± 1.8	2	IV	50 mL vehicle solution containing human serum albumin and heparin	NR
2021	Dilogo et al.	UC	allogenic	NR	Positive: CD90, CD73 Negative: CD34	5–6	NR	1X10^6^	NR	1	IV	100 ml 0.9% saline solution	NR
2021	Adas et al.	UC	allogenic	NR	NR	4	NR	3 X 10^6^	NR	3	IV	NR	NR

UC, umbilical cord; P, passage; F, frequency

ISCT^*^: Met all three criteria (1, 2, and 3 below)

1. Plastic adherence

2. Trilineage differentiation

3. Positive/negative surface markers

### Primary outcome: efficacy

#### Mortality

**Fig. 3:**
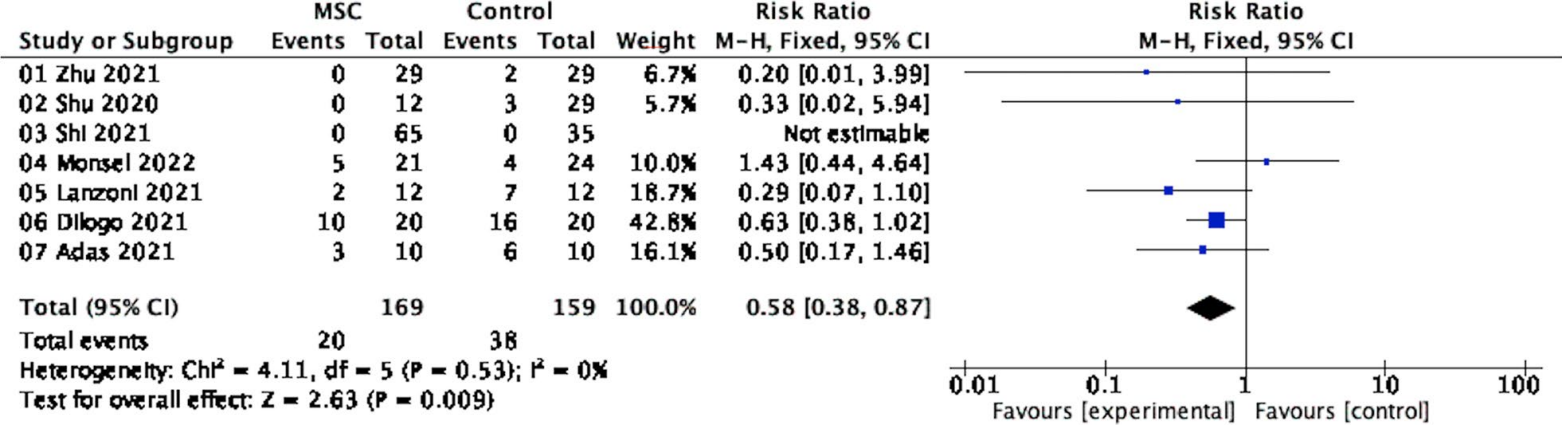
Forest plot showing the difference in the effect of MSC therapy and control treatment on the mortality rate. CI, confidence interval; M–H, Mantel–Haenszel method; Chi^2^, Chi-Squared Test; df, degrees of freedom; I^2^, I-squared statistic

Seven studies including 328 patients with COVID-19 pneumonia reported the mortality incidence (Fig. 3). The mortality rates were 20 of 169 patients in the intervention group and 38 of 159 in the control group. A fixed-effects meta-analysis demonstrated that MSC infusion was associated with a lower mortality risk (RR = 0.58; 95% CI = 0.38, 0.87; P = 0.53; I^2^ = 0%).

### Immune biomarkers

#### CRP

**Fig. 4:**
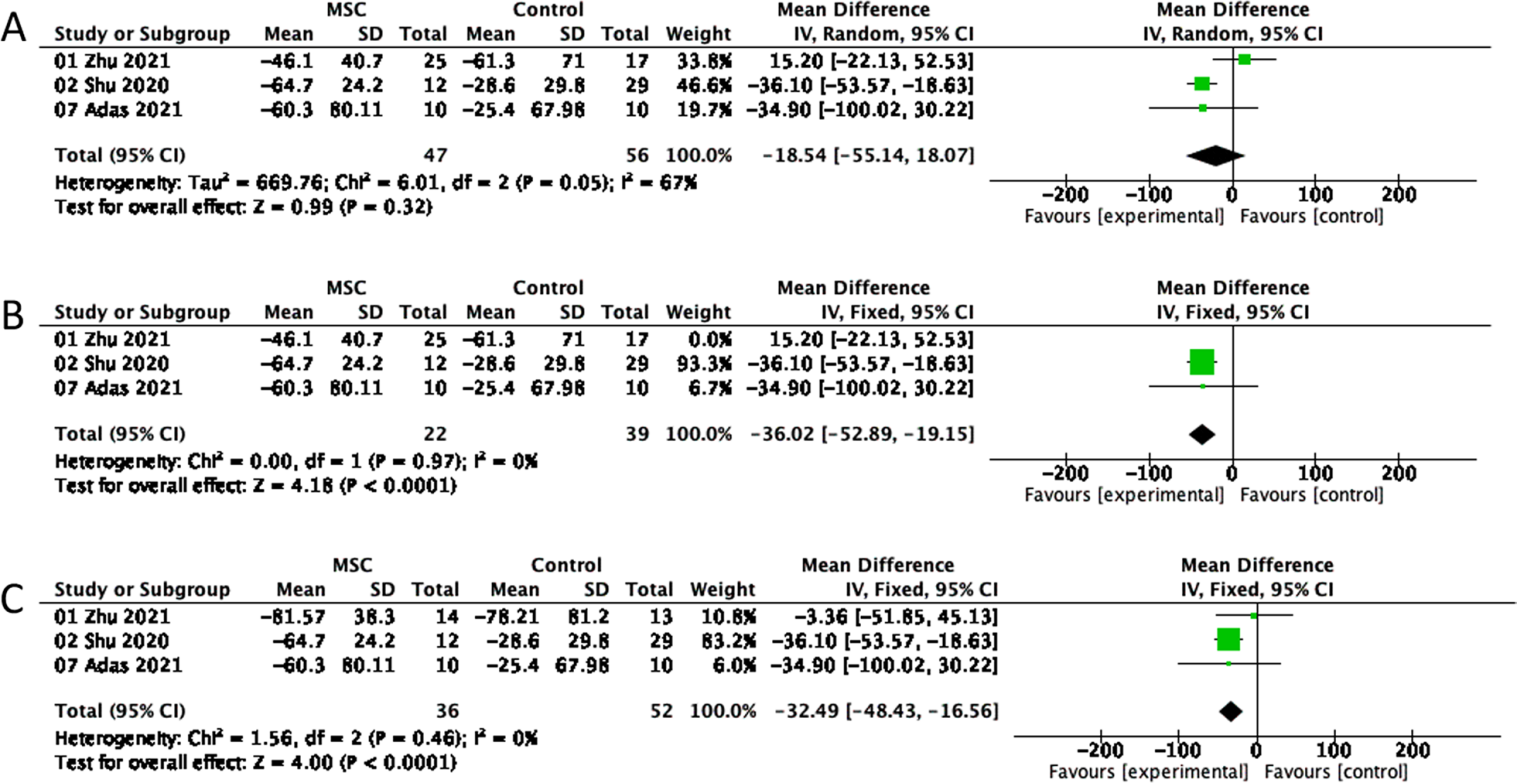
Forest plot depicting variations in CRP in MSC and control groups 7 days after injection. **A** CRP. **B** The sensitivity analysis for CRP. **C** CRP in the severe/critical range of COVID-19 patients. CI, confidence interval; IV, Inverse-variance method; Chi^2^, Chi-Squared Test; df, degrees of freedom; I^2^, I-squared statistic; Tau^2^, Tau-squared statistic

In three studies including 103 participants, changes in CRP levels from baseline to 7 days were assessed in the MSC and control groups. A meta-analysis using a random-effects model revealed no significant CRP changes (Fig. 4A) between the two groups and a degree of heterogeneity (MD =  − 18.54; 95% CI =  − 55.14, 18.07; P = 0.05; I^2^ = 67%). By skipping the sensitivity analysis of each study in succession, we noticed that, when Zhu (2021) was eliminated, the pooled MD and 95% CI were altered and the sample heterogeneity was greatly reduced. In other words, a fixed-effects model meta-analysis revealed that the degree of the CRP decrease (Fig. 4B) was more significant in the MSC group than in the control group (MD =  − 36.02, 95% CI =  − 52.89, − 19.15; P = 0.97; I^2^ = 0%). Of the seven studies, only Zhu (2021) included patients with moderate/mild COVID-19, while Shu (2020) and Adas (2021) included only patients with severe/critical COVID-19. In light of this, the heterogeneity may be due to the varying severity of the patients’ disease. In addition, in the MSC and control groups, Zhu (2021) showed CRP variations at baseline and on day 7 in COVID-19 patients only with severe/critical. The fixed-effects model meta-analysis indicated that the MSC-injected patients were identical. CRP levels decreased (Fig. 4C) more significantly than in the control group (MD =  − 32.49; 95% CI =  − 48.43, − 16.56; P = 0.46; I^2^ = 0%).

#### IFN-γ

**Fig. 5:**
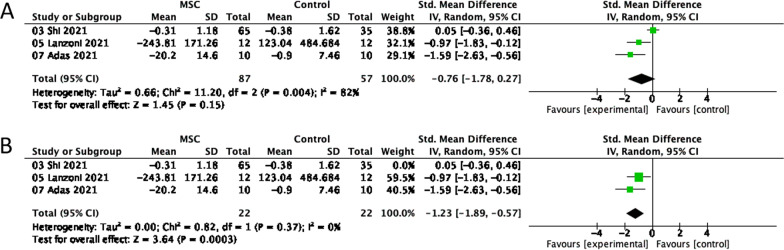
Forest plot illustrating the differences in IFN-γ levels between the MSC and control groups 7 days after injection. **A** IFN-γ. **B** The sensitivity analysis for IFN-γ. CI, confidence interval; IV, Inverse-variance method; Chi^2^, Chi-Squared Test; df, degrees of freedom; I^2^, I-squared statistic; Tau^2^, Tau-squared statistic

Three studies with 144 people assessed changes in IFN-γ from baseline to 1 week. All participants were severe and critical COVID-19 patients. To include the values in the meta-analysis, we converted the IFN-γ median and quartile provided by Lanzoni (2021) to mean and standard deviation. The meta-analysis used a random-effects model, and the level of change in IFN-γ (Fig. 5A) was not significantly different between the two groups (SMD =  − 0.76, 95% CI =  − 1.78, 0.27; P = 0.004; I^2^ = 82%). We performed a sensitivity analysis and found that omitting Shi (2021) significantly reduced heterogeneity and altered the findings: IFN-γ fell more in the MSC group (Fig. 5B), and the results were significantly different (SMD =  − 1.23; 95% CI =  − 1.89, − 0.57; P = 0.37; I^2^ = 0%).

#### IL-6

**Fig. 6:**
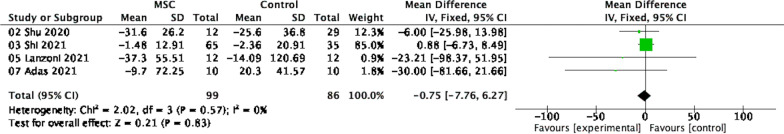
Forest plot showing changes in IL-6 in MSC and control groups 7 days after injection. CI, confidence interval; IV, Inverse-variance method; Chi^2^, Chi-Squared Test; df, degrees of freedom; I^2^, I-squared statistic

Four studies covering 185 subjects without mild COVID-19 assessed the difference in IL-6 between the MSC and control groups from baseline to about a week (Fig. 6). Lanzoni (2021) provided the median and interquartile range, which we converted to mean and standard deviation so that these values could be entered in the meta-analysis. Our results showed that the level of IL-6 was not significantly different between the MSC and control groups (MD =  − 0.75; 95% CI =  − 7.76, 6.27; P = 0.57; I^2^ = 0%).

### Secondary outcomes: safety

#### AEs and SAEs

**Fig. 7:**
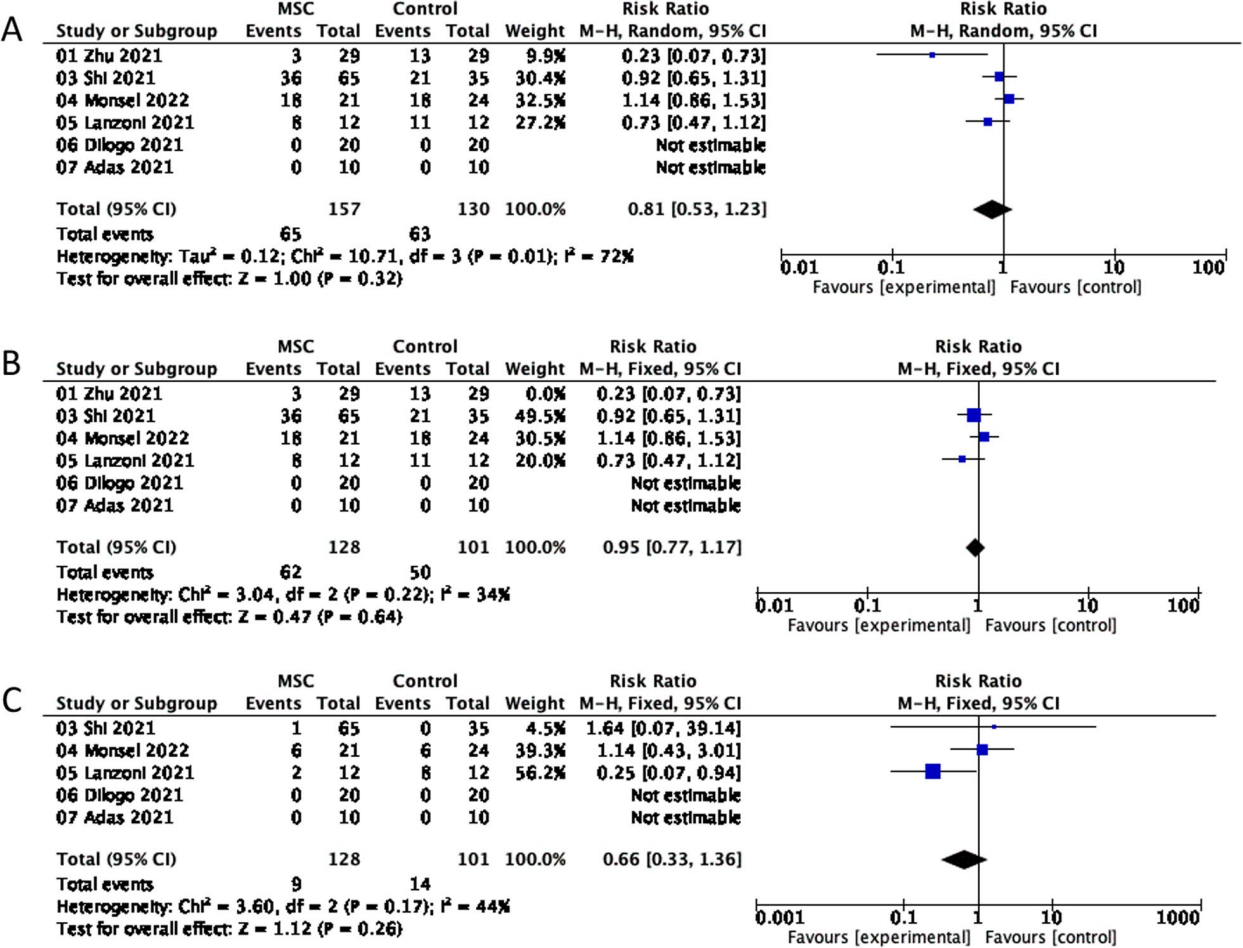
A comparison of the effect of MSC therapy vs. conventional treatment on adverse events and significant adverse events displayed as a forest plot. **A** Adverse events. **B** The sensitivity analysis for adverse events. **C** Serious adverse events. CI, confidence interval; M–H, Mantel–Haenszel method; Chi^2^, Chi-Squared Test; df, degrees of freedom; I^2^, I-squared statistic; Tau^2^, Tau-squared statistic

Six clinical studies reported AEs during therapy (Fig. 7A). The most common AEs were fever, diarrhea, cough, palpitations, alteration of consciousness, pulmonary edema, hypokalemia, raised blood pressure, elevated alanine aminotransferase, elevated aspartate aminotransferase, and elevated lactic acid dehydrogenase. A similar percentage of individuals had AEs in the MSC and control groups (42% vs. 48%). A random-effects meta-analysis revealed no connection between MSC use and AE risk (RR = 0.81; 95% CI = 0.53, 1.23; P = 0.01; I^2^ = 72%). We conducted a sensitivity analysis by eliminating all studies one-by-one (Fig. 7B). The heterogeneity was reduced by excluding Zhu (2021), but the overall impact remained the same (RR = 0.95; 95% CI = 0.77, 1.17; P = 0.22; I^2^ = 34%).

Five studies including 229 individuals analyzed the incidence of SAEs (Fig. 7C). Similarly, the results of a meta-analysis using a fixed-effects model revealed that the incidence of SAEs was marginally lower in the MSC group than in the control group, but the difference was not significant (RR = 0.66; 95% CI = 0.33, 1.36; P = 0.17; I^2^ = 44%).

## Discussion

We conducted a meta-analysis of seven published RCTs that used MSCs to treat COVID-19 pneumonia. We aggregated six outcomes, namely, four indicators for efficacy—mortality, CRP, IFN-γ, and IL-6—and two for safety—AEs and SAEs.

Our data suggest that MSCs are safe and effective in reducing the risk of death compared with standard treatment and placebo. In severe and critical patients, CRP and IFN-γ fell more in the MSC group than in the control group, but IL-6 was largely unchanged.

When evaluating the potential efficacy of a treatment for severe disease, one of the first and most crucial questions to consider is the ability to reduce mortality. According to our meta-analysis, MSC treatment can reduce the risk of death in patients with COVID-19 pneumonia. Other studies show the same outcomes as ours: Kirkham et al. focused on COVID-19 patients who had been treated with MSC-derived products, and their meta-analysis contained four controlled studies with 93 individuals. They identified a significant drop in mortality. Qu et al. conducted a systematic review and meta-analysis of published outcomes of MSC treatment of ARDS patients. While the differences between the control and intervention groups were not significant, they suggested that MSC treatment could effectively reduce mortality.

When many inflammatory factors are released in the body, patients infected with SARS-CoV-2 experience a powerful and uncontrollable immunological response. Fever, weakness, joint discomfort, and muscular soreness are possible symptoms in mild cases. In severe cases, ARDS, septic shock, problems correcting metabolic acidosis, coagulation malfunction, and MOSF can occur. IL-6 is critically involved in the COVID-19-induced cytokine storm and has been linked to the occurrence and progression of COVID-19 pneumonia in several investigations. Unusually high levels of IL-6 have been associated with poor clinical outcomes [43]. Some studies demonstrated that the use of IL-6 blocking agents reduced the risk of poor outcomes and secondary infection in hospitalized COVID-19 patients and was linked to decreased 28-day all-cause mortality and a shorter hospital stay [44–46]. However, other studies have found that these drugs did not shorten the course of the disease, improve respiratory failure, or enhance clinical results versus standard treatment or placebo alone [47, 48]. As a result, we can safely deduce that the role of MSCs in the treatment of COVID-19 pneumonia is potentially reflected in IL-6. Four of the seven RCTs included 185 individuals with IL-6 measurements that could be extracted and combined. The findings revealed that, while there was no difference between the intervention and control groups, there was a trend toward improvement. MSCs were observed to lower IL-6 levels in the Kirkham (2022) meta-analysis. Kirkham (2022) may have included one non-RCT among the three controlled trials, which may explain the variance in results. Secondly, they chose the IL-6 endpoint value, whereas we decided to use the IL-6 difference value. Regardless of whether our findings or their findings are correct, we can detect a positive trend for MSC-induced lowering of IL-6 levels. We eagerly await the analysis of more samples and further clinical trials to determine if MSCs can reduce IL-6 levels in COVID-19 pneumonia patients.

A meta-analysis evaluating laboratory indicators of COVID-19 found that nearly two-thirds of patients had elevated CRP [49]. In addition, elevated CRP may be related to the virus-induced cytokine storm in the body [50]. CRS can be suspected if patients experience rapid respiratory deterioration combined with high fever and disproportionally high CRP and serum ferritin. A total of 103 people in three studies were evaluated for CRP differences before and after treatment measures. Subsequent pooled index analysis found that there was no significant difference in CRP changes between the intervention and control groups. Nonetheless, there was a trend for improvement, with some degree of heterogeneity. In the previous analysis of AEs, we mentioned that sensitivity analysis revealed that this heterogeneity may come from Zhu (2021). This heterogeneity is due to the difference in the included population and blinding of the Zhu (2021) study and differences in implementation objects. However, Zhu (2021) also separately analyzed the changes in CRP in severe/critical patients in the MSC and control groups before and after treatment. We incorporated these stratified data into the other two studies. We found that, after removing some mild/moderate patients, MSCs showed therapeutic potential to reduce CRP levels with less heterogeneity. This result may be related to MSC-mediated alleviation of the cytokine storm. Qu (2020) examined cytokines and inflammatory markers. They also found that levels of the proinflammatory cytokine CRP decreased considerably within 5 days of MSC treatment. In addition, Ghahramani et al. conducted a meta-analysis of inflammatory markers in severe and non-severe COVID-19 patients [51]. They found that, compared with the non-severe group, the inflammatory markers were significantly increased in the severe group. We also saw that CRP was significantly lower in the intervention group than in the control group, excluding mild COVID-19, suggesting that MSCs are a more suitable therapeutic method for severe COVID-19 patients.

Another indicator, IFN-γ, was mentioned in three articles. IFN-γ or type II IFN is an essential proinflammatory molecule in the anti-virus phase of the cytokine storm, especially in virus-induced sepsis [52]. A meta-analysis of the three studies revealed no significant difference between the intervention and control groups and considerable heterogeneity. This result may be due to the patients’ different stages of disease development. When we removed Shi (2021), the results showed that IFN-γ was significantly decreased in the MSC group and that the I^2^ was 0%. Whether MSCs can reduce IFN-γ needs further exploration through studies with larger sample sizes and more prolonged event monitoring.

Safety must be evaluated before considering whether a trial can be conducted with a large sample size for clinical use. Six of the seven studies investigated AEs in 229 people during the experimental phase. The remaining trial (Shu 2020), which did not explore AEs, revealed no adverse reaction in the MSC and control groups. We pooled the results of these studies and found that the incidence of AEs was similar in the two groups and that MSC injection did not increase the risk of AEs compared with the control group. Zhu (2021) was eliminated due to the variability among the six included studies. This significantly reduced the heterogeneity, with the I^2^ decreasing from 72 to 34%. The results of the Zhu trial (2021) (N = 58) showed that only three patients treated with MSCs (n = 29) had AEs, compared with 13 patients in the control group (n = 29). The MSC group had fewer AEs than the control group. The following reasons may underlie this heterogeneity. First, the characteristics of the patients included are slightly different from those of the other studies. Moreover, Zhu (2021) enrolled 15 patients with mild/moderate COVID-19 pneumonia in the MSC group and 16 such patients in the control group, while the remaining five studies only included severe/critical COVID-19 pneumonia patients. In addition, each experimental study had various definitions of the symptoms included as AEs. Hence, the breadth of AEs in these groups varied. The other three RCTs (Shi 2021, Mouse 2022, and Lanzoni 2021), which were estimable, had more AE items relative to Zhu (2021). Finally, although Zhu (2021) did not disclose the grouping information to the participants, they did not blind the investigators or the outcome assessors, unlike the other studies. Because some AEs are subjective outcome measures, implementation and measurement biases are risks. After the sensitivity analysis, even with the exclusion of Zhu (2021), the test results remained stable and there was still no significant difference in AEs between the MSC and control groups.

Only five studies documented SAEs. There was no significant difference between the two groups in the occurrence of serious AEs. Only one patient in the intervention group in Shi (2021) developed pneumothorax, a grade 3 AE unrelated to therapy, while no SAE occurred in the control group. All prespecified infusion-associated AEs in the control group of Lanzoni (2021) happened in the same person, who died of cardiac arrest 2 h after receiving the vehicle solution. MSC infusion had no harmful effect on SAE occurrence compared to the control group. It is worth noting that none of the trials included in this review found any SAE linked to umbilical cord-derived MSC injection. Regarding safety, MSC therapy has a strong track record, and previous clinical studies have proven that it is safe [53–58].

The following features indicate the limitations of our study. First, large-scale RCTs are lacking, and each experimental team has its own criteria for selecting and evaluating outcome indicators. As a result, fewer experimental outcomes could be merged. Besides safety AEs and SAEs, mortality and cytokine levels also reflect efficacy, but our meta-analysis lack more apparent pulmonary function findings, changes in symptoms, and imaging differences. Although we performed a sensitivity analysis and used a random-effects model, it was challenging to achieve a subgroup analysis focusing on the reasons for the heterogeneity due to the small number of included studies. Second, all MSCs in the seven studies were obtained from the umbilical cord. Only three studies stated whether or not the MSCs used in the trial met the basic requirements of the ISCT. As a result, the extension of these data to other types of MSCs should be done with caution. Finally, it is difficult to target patient severity, baseline therapy, and the combination of intervention methods, dose, and single or several injections by subgroup due to the scarcity of RCT research.

Numerous scholars believe that extracellular vesicles (EVs) have considerable potential as an alternative to MSCs. EVs, released by MSCs through paracrine mechanisms, are bilayer membrane structures that deliver bioactive components to target cells. EVs can be classified into three types: microvesicles, exosomes, and apoptotic bodies [59]. MSC-derived EVs have more advantages over their parent cells. First, EVs cannot self-replicate and have low immunogenicity and are thus safer than MSCs. Secondly, EVs are suitable for intranasal or inhalation administration. This method is more efficient than intravenous injection [60]. Finally, many therapeutic substances can be loaded into EVs. However, in practical application, EVs also face numerous challenges. Subgroups of EVs are heterogeneous, but no uniform standard distinguishes them [61]. Mass production of EVs is also an additional concern. In 2014, Zhu et al. found that MSC-derived EVs could alleviate the symptoms of *E. coli* endotoxin-induced acute lung injury in mice [62]. Since then, other researchers have reached similar conclusions and have identified a variety of substances contained in EVs, especially microRNA [63]. Morrison and colleagues also found that MSCs modulate macrophages in a lung injury model by transferring mitochondria [64]. With the outbreak of COVID-19, two studies initially verified the safety and efficacy of MSC-derived exosomes from the veins and airways in severe COVID-19, but the considerable potential of MSC-derived EVs will be revisited in future experiments [65, 66].

Several preclinical studies have explored the pathway(s) of MSCs in the treatment of lung injury, providing new perspectives for clinical research. Some clinical researchers have also recently achieved exciting results. However, there are specific difficulties to consider with this MSC-based strategy for treating COVID-19. For instance, there is currently no uniform standard for the use of MSCs. In addition, the types of COVID-19 patients selected by various research groups also vary. These problems require confirmation in more extensive RCTs and can be solved in other trials.

## Conclusion

Our findings suggest that UC-MSC treatment can reduce inflammatory factors and the probability of death in patients without causing significant AEs. However, before conducting large-scale clinical trials, we must first determine the type and contraindications of COVID-19 pneumonia patients, the source and dosage of MSCs, the need for single or multiple doses, and the administration route and compliance with ISCT minimum standards, among other considerations. As a result, the MSC therapy regimen for COVID-19 pneumonia should be developed further, and its efficacy and safety should be assessed in larger RCTs.

## Supplementary Information

Additional file 1: Table S1. Search phrases for PubMed.

Additional file 2. Table S2. Search phrases for Embase.

Additional file 3. Table S3. Search phrases for Web of Science.

Additional file 4. Table S4. Search phrases for the Cochrane Library.

Additional file 5. Table S5. Summary of included patients.

## Data Availability

The datasets used and analyzed during the current study are available from the corresponding author on reasonable request.

## Abbreviations

MSCs: Mesenchymal stem/stromal cells
UC: Umbilical Cord
COVID-19: Coronavirus disease-19
SARS-CoV-2: Severe Acute Respiratory Syndrome Coronavirus 2
ARDS: Acute respiratory distress syndrome
MOSF: Multiple organ system failure
ACE2: Angiotensin-converting enzyme-2
CRS: Cytokine release syndrome
TMPRSS2: Transmembrane serine protease 2
Revman: Review Manager
AE: Adverse event
SAE: Serious adverse event
IL-2: Interleukin-2
IFN-γ: Interferon-gamma
CRP: C-reaction protein
RR: Relative risk
MD: Mean difference
SMD: Standardized mean difference
95% CI: 95% Confidence interval
RCTs: Randomized controlled trials
ISCT: International Society for Cell & Gene Therapy
RoB: Risk of Bias Tool
EVs: Extracellular vesicles
MVs: Microvesicles
