# Supplementary material for: Efficacy and safety of human umbilical cord-derived mesenchymal stem cells for COVID-19 pneumonia: a meta-analysis of randomized controlled trials
Source: Stem Cell Res Ther. 2023 May 4;14:118. doi: 10.1186/s13287-023-03286-8 (PMC10159228; doi:10.1186/s13287-023-03286-8)
Supplement: Supplementary file 1 — Additional file 1: Table S1. Search phrases for PubMed. [file 13287_2023_3286_MOESM1_ESM.docx]

**Table S1.** search phrases for PubMed

| Search number | Query | Sort By | Filters | Search Details | Results | Time |
| --- | --- | --- | --- | --- | --- | --- |
| 41 | #29 AND #40 |  |  | ("Acute Lung Injury"[MeSH Terms] OR "Respiratory Distress Syndrome"[MeSH Terms] OR (("respirat*"[Title/Abstract] OR "ventilat*"[Title/Abstract]) AND ("distress*"[Title/Abstract] OR "depress*"[Title/Abstract] OR "failure*"[Title/Abstract] OR "insufficien*"[Title/Abstract] OR "paraly*"[Title/Abstract])) OR (("pulmonary*"[Title/Abstract] OR "lung*"[Title/Abstract] OR "alveol*"[Title/Abstract]) AND ("collapse*"[Title/Abstract] OR "injur*"[Title/Abstract] OR "failure*"[Title/Abstract] OR "damage*"[Title/Abstract] OR "shock"[Title/Abstract])) OR (("acute"[Title/Abstract] OR "serious"[Title/Abstract] OR "severe"[Title/Abstract]) AND ("hypox*"[Title/Abstract] OR "respirat*"[Title/Abstract])) OR ("acute"[Title/Abstract] AND ("lung injur*"[Title/Abstract] OR "distress syndrome*"[Title/Abstract])) OR ("ALI"[Title/Abstract] OR "ARDS"[Title/Abstract]) OR ("Critical Illness"[MeSH Terms] OR "Pneumonia"[MeSH Terms] OR "pneumoni*"[Title/Abstract] OR (("lung*"[Title/Abstract] OR "pulmonary*"[Title/Abstract]) AND ("inflammat*"[Title/Abstract] OR "infect*"[Title/Abstract])) OR ("moderate*"[All Fields] OR "common*"[All Fields] OR "severe*"[All Fields] OR "critical*"[All Fields]))) AND ("COVID-19"[MeSH Terms] OR "SARS-CoV-2"[MeSH Terms] OR ("COVID19"[Title/Abstract] OR "2019nCoV"[Title/Abstract] OR "nCoV2019"[Title/Abstract] OR "19nCoV"[Title/Abstract] OR "ncov19"[Title/Abstract]) OR (("2019"[Title/Abstract] OR "19"[Title/Abstract]) AND ("nCoV"[Title/Abstract] OR "novel cov"[Title/Abstract] OR "COVID"[Title/Abstract] OR "coronavir*"[Title/Abstract] OR "corona vir*"[Title/Abstract])) OR (("New"[Title/Abstract] OR "novel"[Title/Abstract]) AND ("coronavir*"[Title/Abstract] OR "corona vir*"[Title/Abstract])) OR ("wuhan"[Title/Abstract] AND ("disease"[Title/Abstract] OR "virus"[Title/Abstract] OR "coronavir*"[Title/Abstract] OR "corona vir*"[Title/Abstract])) OR ("SARS-CoV-2"[Title/Abstract] OR "SARS-CoV2"[Title/Abstract] OR "SARSCoV2"[Title/Abstract] OR "SARSCoV-2"[Title/Abstract]) OR ("SARS coronavirus 2"[Title/Abstract] OR "SARS-like coronavirus"[Title/Abstract] OR "Severe Acute Respiratory Syndrome Coronavirus-2"[Title/Abstract])) AND ("Mesenchymal Stem Cells"[MeSH Terms] OR ("mesenchymal stem cell*"[Title/Abstract] OR "wharton jelly cell*"[Title/Abstract]) OR (("mesenchymal"[Title/Abstract] OR "adipose"[Title/Abstract] OR "marrow"[Title/Abstract] OR "placenta*"[Title/Abstract] OR "umbilical cord"[Title/Abstract] OR "Dental pulp"[Title/Abstract] OR "Wharton's Jelly"[Title/Abstract]) AND ("stem"[Title/Abstract] OR "stroma*"[Title/Abstract] OR "progenitor*"[Title/Abstract]) AND "cell*"[Title/Abstract]) OR (("multipotent"[Title/Abstract] OR "multi-potent"[Title/Abstract]) AND ("stem"[Title/Abstract] OR "stroma*"[Title/Abstract]) AND "cell*"[Title/Abstract]) OR ("MSC"[Title/Abstract] OR "AD-MSC"[Title/Abstract] OR "AT-MSC"[Title/Abstract] OR "ADSC"[Title/Abstract] OR "UC-MSC"[Title/Abstract] OR "WJ-MSC"[Title/Abstract] OR "BM-MSC"[Title/Abstract] OR "DP-MSC"[Title/Abstract] OR "PL-MSC"[Title/Abstract] OR "DSC"[Title/Abstract] OR "hMSC"[Title/Abstract])) AND (("randomized controlled trial"[Publication Type] OR "controlled clinical trial"[Publication Type] OR "randomized"[Title/Abstract] OR "placebo"[Title/Abstract] OR "drug therapy"[MeSH Subheading] OR "randomly"[Title/Abstract] OR "trial"[Title/Abstract] OR "groups"[Title/Abstract]) NOT ("animals"[MeSH Terms] NOT "humans"[MeSH Terms])) | 123 | 03:42:26 |
| 40 | #38 NOT #39 |  |  | ("randomized controlled trial"[Publication Type] OR "controlled clinical trial"[Publication Type] OR "randomized"[Title/Abstract] OR "placebo"[Title/Abstract] OR "drug therapy"[MeSH Subheading] OR "randomly"[Title/Abstract] OR "trial"[Title/Abstract] OR "groups"[Title/Abstract]) NOT ("animals"[MeSH Terms] NOT "humans"[MeSH Terms]) | 4,678,603 | 03:40:40 |
| 39 | animals [mh] NOT humans [mh] |  |  | "animals"[MeSH Terms] NOT "humans"[MeSH Terms] | 4,976,093 | 03:40:27 |
| 38 | #30 OR #31 OR #32 OR #33 OR #34 OR #35 OR #36 OR #37 |  |  | "randomized controlled trial"[Publication Type] OR "controlled clinical trial"[Publication Type] OR "randomized"[Title/Abstract] OR "placebo"[Title/Abstract] OR "drug therapy"[MeSH Subheading] OR "randomly"[Title/Abstract] OR "trial"[Title/Abstract] OR "groups"[Title/Abstract] | 5,370,705 | 03:40:22 |
| 37 | groups [tiab] |  |  | "groups"[Title/Abstract] | 2,358,077 | 03:39:22 |
| 36 | trial [tiab] |  |  | "trial"[Title/Abstract] | 697,813 | 03:39:08 |
| 35 | randomly [tiab] |  |  | "randomly"[Title/Abstract] | 379,240 | 03:39:02 |
| 34 | drug therapy [sh] |  |  | "drug therapy"[MeSH Subheading] | 2,460,015 | 03:38:55 |
| 33 | placebo [tiab] |  |  | "placebo"[Title/Abstract] | 233,025 | 03:38:51 |
| 32 | randomized [tiab] |  |  | "randomized"[Title/Abstract] | 603,751 | 03:38:48 |
| 31 | controlled clinical trial [pt] |  |  | "controlled clinical trial"[Publication Type] | 653,268 | 03:38:44 |
| 30 | randomized controlled trial [pt] |  |  | "randomized controlled trial"[Publication Type] | 563,403 | 03:38:41 |
| 29 | #22 AND #28 |  |  | ("Acute Lung Injury"[MeSH Terms] OR "Respiratory Distress Syndrome"[MeSH Terms] OR (("respirat*"[Title/Abstract] OR "ventilat*"[Title/Abstract]) AND ("distress*"[Title/Abstract] OR "depress*"[Title/Abstract] OR "failure*"[Title/Abstract] OR "insufficien*"[Title/Abstract] OR "paraly*"[Title/Abstract])) OR (("pulmonary*"[Title/Abstract] OR "lung*"[Title/Abstract] OR "alveol*"[Title/Abstract]) AND ("collapse*"[Title/Abstract] OR "injur*"[Title/Abstract] OR "failure*"[Title/Abstract] OR "damage*"[Title/Abstract] OR "shock"[Title/Abstract])) OR (("acute"[Title/Abstract] OR "serious"[Title/Abstract] OR "severe"[Title/Abstract]) AND ("hypox*"[Title/Abstract] OR "respirat*"[Title/Abstract])) OR ("acute"[Title/Abstract] AND ("lung injur*"[Title/Abstract] OR "distress syndrome*"[Title/Abstract])) OR ("ALI"[Title/Abstract] OR "ARDS"[Title/Abstract]) OR ("Critical Illness"[MeSH Terms] OR "Pneumonia"[MeSH Terms] OR "pneumoni*"[Title/Abstract] OR (("lung*"[Title/Abstract] OR "pulmonary*"[Title/Abstract]) AND ("inflammat*"[Title/Abstract] OR "infect*"[Title/Abstract])) OR ("moderate*"[All Fields] OR "common*"[All Fields] OR "severe*"[All Fields] OR "critical*"[All Fields]))) AND ("COVID-19"[MeSH Terms] OR "SARS-CoV-2"[MeSH Terms] OR ("COVID19"[Title/Abstract] OR "2019nCoV"[Title/Abstract] OR "nCoV2019"[Title/Abstract] OR "19nCoV"[Title/Abstract] OR "ncov19"[Title/Abstract]) OR (("2019"[Title/Abstract] OR "19"[Title/Abstract]) AND ("nCoV"[Title/Abstract] OR "novel cov"[Title/Abstract] OR "COVID"[Title/Abstract] OR "coronavir*"[Title/Abstract] OR "corona vir*"[Title/Abstract])) OR (("New"[Title/Abstract] OR "novel"[Title/Abstract]) AND ("coronavir*"[Title/Abstract] OR "corona vir*"[Title/Abstract])) OR ("wuhan"[Title/Abstract] AND ("disease"[Title/Abstract] OR "virus"[Title/Abstract] OR "coronavir*"[Title/Abstract] OR "corona vir*"[Title/Abstract])) OR ("SARS-CoV-2"[Title/Abstract] OR "SARS-CoV2"[Title/Abstract] OR "SARSCoV2"[Title/Abstract] OR "SARSCoV-2"[Title/Abstract]) OR ("SARS coronavirus 2"[Title/Abstract] OR "SARS-like coronavirus"[Title/Abstract] OR "Severe Acute Respiratory Syndrome Coronavirus-2"[Title/Abstract])) AND ("Mesenchymal Stem Cells"[MeSH Terms] OR ("mesenchymal stem cell*"[Title/Abstract] OR "wharton jelly cell*"[Title/Abstract]) OR (("mesenchymal"[Title/Abstract] OR "adipose"[Title/Abstract] OR "marrow"[Title/Abstract] OR "placenta*"[Title/Abstract] OR "umbilical cord"[Title/Abstract] OR "Dental pulp"[Title/Abstract] OR "Wharton's Jelly"[Title/Abstract]) AND ("stem"[Title/Abstract] OR "stroma*"[Title/Abstract] OR "progenitor*"[Title/Abstract]) AND "cell*"[Title/Abstract]) OR (("multipotent"[Title/Abstract] OR "multi-potent"[Title/Abstract]) AND ("stem"[Title/Abstract] OR "stroma*"[Title/Abstract]) AND "cell*"[Title/Abstract]) OR ("MSC"[Title/Abstract] OR "AD-MSC"[Title/Abstract] OR "AT-MSC"[Title/Abstract] OR "ADSC"[Title/Abstract] OR "UC-MSC"[Title/Abstract] OR "WJ-MSC"[Title/Abstract] OR "BM-MSC"[Title/Abstract] OR "DP-MSC"[Title/Abstract] OR "PL-MSC"[Title/Abstract] OR "DSC"[Title/Abstract] OR "hMSC"[Title/Abstract])) | 520 | 03:38:27 |
| 28 | #23 OR #24 OR #25 OR #26 OR #27 |  |  | "Mesenchymal Stem Cells"[MeSH Terms] OR ("mesenchymal stem cell*"[Title/Abstract] OR "wharton jelly cell*"[Title/Abstract]) OR (("mesenchymal"[Title/Abstract] OR "adipose"[Title/Abstract] OR "marrow"[Title/Abstract] OR "placenta*"[Title/Abstract] OR "umbilical cord"[Title/Abstract] OR "Dental pulp"[Title/Abstract] OR "Wharton's Jelly"[Title/Abstract]) AND ("stem"[Title/Abstract] OR "stroma*"[Title/Abstract] OR "progenitor*"[Title/Abstract]) AND "cell*"[Title/Abstract]) OR (("multipotent"[Title/Abstract] OR "multi-potent"[Title/Abstract]) AND ("stem"[Title/Abstract] OR "stroma*"[Title/Abstract]) AND "cell*"[Title/Abstract]) OR ("MSC"[Title/Abstract] OR "AD-MSC"[Title/Abstract] OR "AT-MSC"[Title/Abstract] OR "ADSC"[Title/Abstract] OR "UC-MSC"[Title/Abstract] OR "WJ-MSC"[Title/Abstract] OR "BM-MSC"[Title/Abstract] OR "DP-MSC"[Title/Abstract] OR "PL-MSC"[Title/Abstract] OR "DSC"[Title/Abstract] OR "hMSC"[Title/Abstract]) | 183,463 | 03:38:12 |
| 27 | MSC?[Title/Abstract] OR AD-MSC?[Title/Abstract] OR AT-MSC?[Title/Abstract] OR ADSC?[Title/Abstract] OR UC-MSC?[Title/Abstract] OR WJ-MSC?[Title/Abstract] OR BM-MSC?[Title/Abstract] OR DP-MSC?[Title/Abstract] OR PL-MSC?[Title/Abstract] OR DSC?[Title/Abstract] OR hMSC?[Title/Abstract] |  |  | "MSC"[Title/Abstract] OR "AD-MSC"[Title/Abstract] OR "AT-MSC"[Title/Abstract] OR "ADSC"[Title/Abstract] OR "UC-MSC"[Title/Abstract] OR "WJ-MSC"[Title/Abstract] OR "BM-MSC"[Title/Abstract] OR "DP-MSC"[Title/Abstract] OR "PL-MSC"[Title/Abstract] OR "DSC"[Title/Abstract] OR "hMSC"[Title/Abstract] | 48,176 | 03:36:41 |
| 26 | (multipotent[Title/Abstract] OR multi-potent[Title/Abstract]) AND ( stem[Title/Abstract] OR stroma* [Title/Abstract]) AND cell*[Title/Abstract] |  |  | ("multipotent"[Title/Abstract] OR "multi-potent"[Title/Abstract]) AND ("stem"[Title/Abstract] OR "stroma*"[Title/Abstract]) AND "cell*"[Title/Abstract] | 9,512 | 03:32:02 |
| 25 | (mesenchymal[Title/Abstract] OR adipose[Title/Abstract] OR marrow[Title/Abstract] OR placenta*[Title/Abstract] OR "umbilical cord"[Title/Abstract] OR "Dental pulp"[Title/Abstract] OR "Wharton’s Jelly" [Title/Abstract]) AND (stem[Title/Abstract] OR stroma*[Title/Abstract] OR Progenitor*[Title/Abstract]) AND cell*[Title/Abstract] |  |  | ("mesenchymal"[Title/Abstract] OR "adipose"[Title/Abstract] OR "marrow"[Title/Abstract] OR "placenta*"[Title/Abstract] OR "umbilical cord"[Title/Abstract] OR "Dental pulp"[Title/Abstract] OR "Wharton's Jelly"[Title/Abstract]) AND ("stem"[Title/Abstract] OR "stroma*"[Title/Abstract] OR "progenitor*"[Title/Abstract]) AND "cell*"[Title/Abstract] | 150,632 | 03:31:43 |
| 24 | ("Mesenchymal stem cell*"[Title/Abstract]) OR ("wharton* Jelly cell*"[Title/Abstract]) |  |  | "mesenchymal stem cell*"[Title/Abstract] OR "wharton jelly cell*"[Title/Abstract] | 53,410 | 03:29:45 |
| 23 | "Mesenchymal Stem Cells"[Mesh] |  |  | "Mesenchymal Stem Cells"[MeSH Terms] | 45,942 | 03:29:40 |
| 22 | #21 AND #8 |  |  | ("Acute Lung Injury"[MeSH Terms] OR "Respiratory Distress Syndrome"[MeSH Terms] OR (("respirat*"[Title/Abstract] OR "ventilat*"[Title/Abstract]) AND ("distress*"[Title/Abstract] OR "depress*"[Title/Abstract] OR "failure*"[Title/Abstract] OR "insufficien*"[Title/Abstract] OR "paraly*"[Title/Abstract])) OR (("pulmonary*"[Title/Abstract] OR "lung*"[Title/Abstract] OR "alveol*"[Title/Abstract]) AND ("collapse*"[Title/Abstract] OR "injur*"[Title/Abstract] OR "failure*"[Title/Abstract] OR "damage*"[Title/Abstract] OR "shock"[Title/Abstract])) OR (("acute"[Title/Abstract] OR "serious"[Title/Abstract] OR "severe"[Title/Abstract]) AND ("hypox*"[Title/Abstract] OR "respirat*"[Title/Abstract])) OR ("acute"[Title/Abstract] AND ("lung injur*"[Title/Abstract] OR "distress syndrome*"[Title/Abstract])) OR ("ALI"[Title/Abstract] OR "ARDS"[Title/Abstract]) OR ("Critical Illness"[MeSH Terms] OR "Pneumonia"[MeSH Terms] OR "pneumoni*"[Title/Abstract] OR (("lung*"[Title/Abstract] OR "pulmonary*"[Title/Abstract]) AND ("inflammat*"[Title/Abstract] OR "infect*"[Title/Abstract])) OR ("moderate*"[All Fields] OR "common*"[All Fields] OR "severe*"[All Fields] OR "critical*"[All Fields]))) AND ("COVID-19"[MeSH Terms] OR "SARS-CoV-2"[MeSH Terms] OR ("COVID19"[Title/Abstract] OR "2019nCoV"[Title/Abstract] OR "nCoV2019"[Title/Abstract] OR "19nCoV"[Title/Abstract] OR "ncov19"[Title/Abstract]) OR (("2019"[Title/Abstract] OR "19"[Title/Abstract]) AND ("nCoV"[Title/Abstract] OR "novel cov"[Title/Abstract] OR "COVID"[Title/Abstract] OR "coronavir*"[Title/Abstract] OR "corona vir*"[Title/Abstract])) OR (("New"[Title/Abstract] OR "novel"[Title/Abstract]) AND ("coronavir*"[Title/Abstract] OR "corona vir*"[Title/Abstract])) OR ("wuhan"[Title/Abstract] AND ("disease"[Title/Abstract] OR "virus"[Title/Abstract] OR "coronavir*"[Title/Abstract] OR "corona vir*"[Title/Abstract])) OR ("SARS-CoV-2"[Title/Abstract] OR "SARS-CoV2"[Title/Abstract] OR "SARSCoV2"[Title/Abstract] OR "SARSCoV-2"[Title/Abstract]) OR ("SARS coronavirus 2"[Title/Abstract] OR "SARS-like coronavirus"[Title/Abstract] OR "Severe Acute Respiratory Syndrome Coronavirus-2"[Title/Abstract])) | 184,078 | 03:29:17 |
| 21 | #15 OR #20 |  |  | "Acute Lung Injury"[MeSH Terms] OR "Respiratory Distress Syndrome"[MeSH Terms] OR (("respirat*"[Title/Abstract] OR "ventilat*"[Title/Abstract]) AND ("distress*"[Title/Abstract] OR "depress*"[Title/Abstract] OR "failure*"[Title/Abstract] OR "insufficien*"[Title/Abstract] OR "paraly*"[Title/Abstract])) OR (("pulmonary*"[Title/Abstract] OR "lung*"[Title/Abstract] OR "alveol*"[Title/Abstract]) AND ("collapse*"[Title/Abstract] OR "injur*"[Title/Abstract] OR "failure*"[Title/Abstract] OR "damage*"[Title/Abstract] OR "shock"[Title/Abstract])) OR (("acute"[Title/Abstract] OR "serious"[Title/Abstract] OR "severe"[Title/Abstract]) AND ("hypox*"[Title/Abstract] OR "respirat*"[Title/Abstract])) OR ("acute"[Title/Abstract] AND ("lung injur*"[Title/Abstract] OR "distress syndrome*"[Title/Abstract])) OR ("ALI"[Title/Abstract] OR "ARDS"[Title/Abstract]) OR ("Critical Illness"[MeSH Terms] OR "Pneumonia"[MeSH Terms] OR "pneumoni*"[Title/Abstract] OR (("lung*"[Title/Abstract] OR "pulmonary*"[Title/Abstract]) AND ("inflammat*"[Title/Abstract] OR "infect*"[Title/Abstract])) OR ("moderate*"[All Fields] OR "common*"[All Fields] OR "severe*"[All Fields] OR "critical*"[All Fields])) | 5,188,551 | 03:28:07 |
| 20 | #16 OR #17 OR #18 OR #19 |  |  | "Critical Illness"[MeSH Terms] OR "Pneumonia"[MeSH Terms] OR "pneumoni*"[Title/Abstract] OR (("lung*"[Title/Abstract] OR "pulmonary*"[Title/Abstract]) AND ("inflammat*"[Title/Abstract] OR "infect*"[Title/Abstract])) OR ("moderate*"[All Fields] OR "common*"[All Fields] OR "severe*"[All Fields] OR "critical*"[All Fields]) | 5,011,063 | 03:27:52 |
| 19 | Moderate* OR common* OR severe* OR critical* |  |  | "moderate*"[All Fields] OR "common*"[All Fields] OR "severe*"[All Fields] OR "critical*"[All Fields] | 4,646,768 | 03:27:19 |
| 18 | (Lung*[Title/Abstract] OR pulmonary*[Title/Abstract]) AND (Inflammat*[Title/Abstract] OR infect*[Title/Abstract]) |  |  | ("lung*"[Title/Abstract] OR "pulmonary*"[Title/Abstract]) AND ("inflammat*"[Title/Abstract] OR "infect*"[Title/Abstract]) | 206,577 | 03:27:11 |
| 17 | Pneumoni*[Title/Abstract] |  |  | "pneumoni*"[Title/Abstract] | 209,478 | 03:25:59 |
| 16 | ("Critical Illness"[Mesh]) OR "Pneumonia"[Mesh] | Most Recent |  | "Critical Illness"[MeSH Terms] OR "Pneumonia"[MeSH Terms] | 276,425 | 03:25:23 |
| 15 | #9 OR #10 OR #11 OR #12 OR #13 OR #14 |  |  | "Acute Lung Injury"[MeSH Terms] OR "Respiratory Distress Syndrome"[MeSH Terms] OR (("respirat*"[Title/Abstract] OR "ventilat*"[Title/Abstract]) AND ("distress*"[Title/Abstract] OR "depress*"[Title/Abstract] OR "failure*"[Title/Abstract] OR "insufficien*"[Title/Abstract] OR "paraly*"[Title/Abstract])) OR (("pulmonary*"[Title/Abstract] OR "lung*"[Title/Abstract] OR "alveol*"[Title/Abstract]) AND ("collapse*"[Title/Abstract] OR "injur*"[Title/Abstract] OR "failure*"[Title/Abstract] OR "damage*"[Title/Abstract] OR "shock"[Title/Abstract])) OR (("acute"[Title/Abstract] OR "serious"[Title/Abstract] OR "severe"[Title/Abstract]) AND ("hypox*"[Title/Abstract] OR "respirat*"[Title/Abstract])) OR ("acute"[Title/Abstract] AND ("lung injur*"[Title/Abstract] OR "distress syndrome*"[Title/Abstract])) OR ("ALI"[Title/Abstract] OR "ARDS"[Title/Abstract]) | 435,341 | 03:23:05 |
| 14 | (ALI[Title/Abstract]) OR (ARDS[Title/Abstract]) |  |  | "ALI"[Title/Abstract] OR "ARDS"[Title/Abstract] | 24,115 | 03:22:00 |
| 13 | (acute[Title/Abstract] AND (lung injur*[Title/Abstract] OR distress syndrome*[Title/Abstract])) |  |  | "acute"[Title/Abstract] AND ("lung injur*"[Title/Abstract] OR "distress syndrome*"[Title/Abstract]) | 35,905 | 03:21:56 |
| 12 | (acute[Title/Abstract] OR serious[Title/Abstract] OR severe[Title/Abstract]) AND (hypox*[Title/Abstract] OR respirat*[Title/Abstract]) |  |  | ("acute"[Title/Abstract] OR "serious"[Title/Abstract] OR "severe"[Title/Abstract]) AND ("hypox*"[Title/Abstract] OR "respirat*"[Title/Abstract]) | 201,791 | 03:21:51 |
| 11 | (pulmonary*[Title/Abstract] OR lung*[Title/Abstract] OR alveol*[Title/Abstract]) AND (collapse*[Title/Abstract] OR injur*[Title/Abstract] OR failure*[Title/Abstract] OR damage*[Title/Abstract] OR shock[Title/Abstract]) |  |  | ("pulmonary*"[Title/Abstract] OR "lung*"[Title/Abstract] OR "alveol*"[Title/Abstract]) AND ("collapse*"[Title/Abstract] OR "injur*"[Title/Abstract] OR "failure*"[Title/Abstract] OR "damage*"[Title/Abstract] OR "shock"[Title/Abstract]) | 177,775 | 03:21:38 |
| 10 | (respirat*[Title/Abstract] OR ventilat*[Title/Abstract]) AND (distress*[Title/Abstract] OR depress*[Title/Abstract] OR failure*[Title/Abstract] OR insufficien*[Title/Abstract] OR paraly*[Title/Abstract]) |  |  | ("respirat*"[Title/Abstract] OR "ventilat*"[Title/Abstract]) AND ("distress*"[Title/Abstract] OR "depress*"[Title/Abstract] OR "failure*"[Title/Abstract] OR "insufficien*"[Title/Abstract] OR "paraly*"[Title/Abstract]) | 148,386 | 03:20:50 |
| 9 | ("Acute Lung Injury"[Mesh]) OR "Respiratory Distress Syndrome"[Mesh] |  |  | "Acute Lung Injury"[MeSH Terms] OR "Respiratory Distress Syndrome"[MeSH Terms] | 44,497 | 03:20:25 |
| 8 | #1 OR #2 OR #3 OR #4 OR #5 OR #6 OR #7 |  |  | "COVID-19"[MeSH Terms] OR "SARS-CoV-2"[MeSH Terms] OR ("COVID19"[Title/Abstract] OR "2019nCoV"[Title/Abstract] OR "nCoV2019"[Title/Abstract] OR "19nCoV"[Title/Abstract] OR "ncov19"[Title/Abstract]) OR (("2019"[Title/Abstract] OR "19"[Title/Abstract]) AND ("nCoV"[Title/Abstract] OR "novel cov"[Title/Abstract] OR "COVID"[Title/Abstract] OR "coronavir*"[Title/Abstract] OR "corona vir*"[Title/Abstract])) OR (("New"[Title/Abstract] OR "novel"[Title/Abstract]) AND ("coronavir*"[Title/Abstract] OR "corona vir*"[Title/Abstract])) OR ("wuhan"[Title/Abstract] AND ("disease"[Title/Abstract] OR "virus"[Title/Abstract] OR "coronavir*"[Title/Abstract] OR "corona vir*"[Title/Abstract])) OR ("SARS-CoV-2"[Title/Abstract] OR "SARS-CoV2"[Title/Abstract] OR "SARSCoV2"[Title/Abstract] OR "SARSCoV-2"[Title/Abstract]) OR ("SARS coronavirus 2"[Title/Abstract] OR "SARS-like coronavirus"[Title/Abstract] OR "Severe Acute Respiratory Syndrome Coronavirus-2"[Title/Abstract]) | 239,491 | 03:20:09 |
| 7 | ("SARS coronavirus 2"[Title/Abstract] OR "SARS‐like coronavirus"[Title/Abstract] OR "Severe Acute Respiratory Syndrome Coronavirus‐2"[Title/Abstract]) |  |  | "SARS coronavirus 2"[Title/Abstract] OR "SARS-like coronavirus"[Title/Abstract] OR "Severe Acute Respiratory Syndrome Coronavirus-2"[Title/Abstract] | 23,663 | 03:19:14 |
| 6 | ("SARS‐CoV‐2"[Title/Abstract] OR "SARS‐CoV2"[Title/Abstract] OR SARSCoV2[Title/Abstract] OR "SARSCoV‐2"[Title/Abstract]) |  |  | "SARS-CoV-2"[Title/Abstract] OR "SARS-CoV2"[Title/Abstract] OR "SARSCoV2"[Title/Abstract] OR "SARSCoV-2"[Title/Abstract] | 79,817 | 03:19:10 |
| 5 | wuhan[Title/Abstract] AND (disease[Title/Abstract] OR virus[Title/Abstract] OR coronavir*[Title/Abstract] OR ‘corona vir* ‘[Title/Abstract]) |  |  | "wuhan"[Title/Abstract] AND ("disease"[Title/Abstract] OR "virus"[Title/Abstract] OR "coronavir*"[Title/Abstract] OR "corona vir*"[Title/Abstract]) | 6,325 | 03:19:05 |
| 4 | (New[Title/Abstract] OR novel[Title/Abstract]) AND (coronavir*[Title/Abstract] OR ‘corona vir* ‘[Title/Abstract]) |  |  | ("New"[Title/Abstract] OR "novel"[Title/Abstract]) AND ("coronavir*"[Title/Abstract] OR "corona vir*"[Title/Abstract]) | 29,802 | 03:18:53 |
| 3 | (2019[Title/Abstract] OR 19[Title/Abstract]) AND (nCoV[Title/Abstract] OR novel CoV[Title/Abstract] OR COVID[Title/Abstract] OR coronavir*[Title/Abstract] OR ‘corona vir* ’[Title/Abstract]) |  |  | ("2019"[Title/Abstract] OR "19"[Title/Abstract]) AND ("nCoV"[Title/Abstract] OR "novel cov"[Title/Abstract] OR "COVID"[Title/Abstract] OR "coronavir*"[Title/Abstract] OR "corona vir*"[Title/Abstract]) | 212,983 | 03:18:44 |
| 2 | COVID19[Title/Abstract] OR 2019nCoV[Title/Abstract] OR nCoV2019[Title/Abstract] OR 19nCoV[Title/Abstract] OR ncov19[Title/Abstract] |  |  | "COVID19"[Title/Abstract] OR "2019nCoV"[Title/Abstract] OR "nCoV2019"[Title/Abstract] OR "19nCoV"[Title/Abstract] OR "ncov19"[Title/Abstract] | 197,733 | 03:18:34 |
| 1 | ("COVID-19"[Mesh]) OR "SARS-CoV-2"[Mesh] |  |  | "COVID-19"[MeSH Terms] OR "SARS-CoV-2"[MeSH Terms] | 148,736 | 03:18:28 |
