# Supplementary material for: Efficacy and safety of human umbilical cord-derived mesenchymal stem cells for COVID-19 pneumonia: a meta-analysis of randomized controlled trials
Source: Stem Cell Res Ther. 2023 May 4;14:118. doi: 10.1186/s13287-023-03286-8 (PMC10159228; doi:10.1186/s13287-023-03286-8)
Supplement: Supplementary file 2 — Additional file 2. Table S2. Search phrases for Embase. [file 13287_2023_3286_MOESM2_ESM.docx]

**Table S2.** search phrases for Embase

| No. | Query | Results | Date |
| --- | --- | --- | --- |
| #69 | #33 AND #68 | 82 | 22-Mar-22 |
| #68 | #53 NOT #67 | 5102805 | 22-Mar-22 |
| #67 | #54 OR #55 OR #56 OR #57 OR #58 OR #59 OR #60 OR #61 OR #62 OR #63 OR #65 OR #66 | 3916688 | 22-Mar-22 |
| #66 | 'animal experiment'/de NOT ('human experiment'/de OR 'human'/de) | 2407448 | 22-Mar-22 |
| #65 | (rat:ti,tt OR rats:ti,tt OR mouse:ti,tt OR mice:ti,tt OR swine:ti,tt OR porcine:ti,tt OR murine:ti,tt OR sheep:ti,tt OR lambs:ti,tt OR pigs:ti,tt OR piglets:ti,tt OR rabbit:ti,tt OR rabbits:ti,tt OR cat:ti,tt OR cats:ti,tt OR dog:ti,tt OR dogs:ti,tt OR cattle:ti,tt OR bovine:ti,tt OR monkey:ti,tt OR monkeys:ti,tt OR trout:ti,tt OR marmoset*:ti,tt) AND 'animal experiment'/de | 1147085 | 22-Mar-22 |
| #64 | (databases NEAR/5 searched):ab | 52855 | 22-Mar-22 |
| #63 | 'update review':ab | 121 | 22-Mar-22 |
| #62 | 'we searched':ab AND (review:ti,tt OR review:it) | 40756 | 22-Mar-22 |
| #61 | review:ab AND review:it NOT trial:ti,tt | 962022 | 22-Mar-22 |
| #60 | ('random cluster' NEAR/4 sampl*):ti,ab,tt | 1534 | 22-Mar-22 |
| #59 | 'random field*':ti,ab,tt | 2625 | 22-Mar-22 |
| #58 | nonrandom*:ti,ab,tt NOT random*:ti,ab,tt | 17662 | 22-Mar-22 |
| #57 | 'systematic review':ti,tt NOT (trial:ti,tt OR study:ti,tt) | 203093 | 22-Mar-22 |
| #56 | 'case control*':ti,ab,tt AND random*:ti,ab,tt NOT ('randomised controlled':ti,ab,tt OR 'randomized controlled':ti,ab,tt) | 19496 | 22-Mar-22 |
| #55 | 'cross‐sectional study' NOT ('randomized controlled trial'/de OR 'controlled clinical study'/de OR 'controlled study'/de OR 'randomised controlled':ti,ab,tt OR 'randomized controlled':ti,ab,tt OR 'control group':ti,ab,tt OR 'control groups':ti,ab,tt) | 323812 | 22-Mar-22 |
| #54 | ((random* NEXT/1 sampl* NEAR/8 ('cross section*' OR questionnaire* OR survey OR surveys OR database OR databases)):ti,ab,tt) NOT ('comparative study'/de OR 'controlled study'/de OR 'randomised controlled':ti,ab,tt OR 'randomized controlled':ti,ab,tt OR 'randomly assigned':ti,ab,tt) | 2817 | 22-Mar-22 |
| #53 | #34 OR #35 OR #36 OR #37 OR #38 OR #39 OR #40 OR #41 OR #42 OR #43 OR #44 OR #45 OR #46 OR #47 OR #48 OR #49 OR #50 OR #51 OR #52 | 5745453 | 22-Mar-22 |
| #52 | trial:ti,tt | 359831 | 22-Mar-22 |
| #51 | 'human experiment'/de | 570935 | 22-Mar-22 |
| #50 | volunteer:ti,ab,tt OR volunteers:ti,ab,tt | 267512 | 22-Mar-22 |
| #49 | (controlled NEAR/8 (study OR design OR trial)):ti,ab,tt | 410810 | 22-Mar-22 |
| #48 | assigned:ti,ab,tt OR allocated:ti,ab,tt | 441652 | 22-Mar-22 |
| #47 | ((assign* OR match OR matched OR allocation) NEAR/6 (alternate OR group OR groups OR intervention OR interventions OR patient OR patients OR subject OR subjects OR participant OR participants)):ti,ab,tt | 413813 | 22-Mar-22 |
| #46 | crossover:ti,ab,tt OR 'cross over':ti,ab,tt | 115595 | 22-Mar-22 |
| #45 | (parallel NEXT/1 group*):ti,ab,tt | 29082 | 22-Mar-22 |
| #44 | 'double blind procedure'/de | 193812 | 22-Mar-22 |
| #43 | ((double OR single OR doubly OR singly) NEXT/1 (blind OR blinded OR blindly)):ti,ab,tt | 256420 | 22-Mar-22 |
| #42 | (open NEXT/1 label):ti,ab,tt | 95144 | 22-Mar-22 |
| #41 | (evaluated:ab OR evaluate:ab OR evaluating:ab OR assessed:ab OR assess:ab) AND (compare:ab OR compared:ab OR comparing:ab OR comparison:ab) | 2461879 | 22-Mar-22 |
| #40 | compare:ti,tt OR compared:ti,tt OR comparison:ti,tt | 583049 | 22-Mar-22 |
| #39 | placebo:ti,ab,tt | 339005 | 22-Mar-22 |
| #38 | 'intermethod comparison'/de | 283059 | 22-Mar-22 |
| #37 | 'randomization'/de | 93342 | 22-Mar-22 |
| #36 | random*:ti,ab,tt | 1766021 | 22-Mar-22 |
| #35 | 'controlled clinical trial'/de | 436489 | 22-Mar-22 |
| #34 | 'randomized controlled trial'/de | 702254 | 22-Mar-22 |
| #33 | #25 AND #32 | 625 | 22-Mar-22 |
| #32 | #26 OR #27 OR #28 OR #29 OR #30 OR #31 | 261505 | 22-Mar-22 |
| #31 | msc*:ti,ab,kw OR 'ad msc?':ti,ab,kw OR 'at msc?':ti,ab,kw OR adsc?:ti,ab,kw OR 'uc msc?':ti,ab,kw OR 'wj msc?':ti,ab,kw OR 'bm msc?':ti,ab,kw OR 'dp msc?':ti,ab,kw OR 'pl msc?':ti,ab,kw OR dsc?:ti,ab,kw OR hmsc?:ti,ab,kw | 78573 | 22-Mar-22 |
| #30 | (multipotent:ti,ab,kw OR 'multi potent':ti,ab,kw) AND (stem:ti,ab,kw OR stroma*:ti,ab,kw) AND cell*:ti,ab,kw | 13445 | 22-Mar-22 |
| #29 | (mesenchymal:ti,ab,kw OR adipose:ti,ab,kw OR marrow:ti,ab,kw OR placenta*:ti,ab,kw OR 'umbilical cord':ti,ab,kw OR 'dental pulp':ti,ab,kw OR 'wharton* jelly':ti,ab,kw) AND (stem:ti,ab,kw OR stroma*:ti,ab,kw OR progenitor*:ti,ab,kw) AND cell*:ti,ab,kw | 227030 | 22-Mar-22 |
| #28 | 'wharton* jelly cell*':ti,ab,kw | 6 | 22-Mar-22 |
| #27 | 'mesenchymal stem cell*':ti,ab,kw | 74709 | 22-Mar-22 |
| #26 | 'mesenchymal stem cell'/exp | 71067 | 22-Mar-22 |
| #25 | #9 AND #24 | 113981 | 22-Mar-22 |
| #24 | #17 OR #23 | 6730370 | 22-Mar-22 |
| #23 | #18 OR #19 OR #20 OR #21 OR #22 | 6455596 | 22-Mar-22 |
| #22 | moderate*:ti,ab,kw OR common*:ti,ab,kw OR severe*:ti,ab,kw OR critical*:ti,ab,kw | 5962980 | 22-Mar-22 |
| #21 | (lung*:ti,ab,kw OR pulmonary*:ti,ab,kw) AND (inflammat*:ti,ab,kw OR infect*:ti,ab,kw) | 315664 | 22-Mar-22 |
| #20 | pneumoni*:ti,ab,kw | 301846 | 22-Mar-22 |
| #19 | 'critical illness'/exp | 32949 | 22-Mar-22 |
| #18 | 'pneumonia'/exp | 370047 | 22-Mar-22 |
| #17 | #10 OR #11 OR #12 OR #13 OR #14 OR #15 OR #16 | 666915 | 22-Mar-22 |
| #16 | ali:ti,ab,kw OR ards:ti,ab,kw | 40027 | 22-Mar-22 |
| #15 | acute:ti,ab,kw AND ('lung injur*':ti,ab,kw OR 'distress syndrome*':ti,ab,kw) | 51191 | 22-Mar-22 |
| #14 | (acute:ti,ab,kw OR serious:ti,ab,kw OR severe:ti,ab,kw) AND (hypox*:ti,ab,kw OR respirat*:ti,ab,kw) | 295714 | 22-Mar-22 |
| #13 | (pulmonary*:ti,ab,kw OR lung*:ti,ab,kw OR alveol*:ti,ab,kw) AND (collapse*:ti,ab,kw OR injur*:ti,ab,kw OR failure*:ti,ab,kw OR damage*:ti,ab,kw OR shock:ti,ab,kw) | 282414 | 22-Mar-22 |
| #12 | (respirat*:ti,ab,kw OR ventilat*:ti,ab,kw) AND (distress*:ti,ab,kw OR depress*:ti,ab,kw OR failure*:ti,ab,kw OR insufficien*:ti,ab,kw OR paraly*:ti,ab,kw) | 231900 | 22-Mar-22 |
| #11 | 'acute lung injury'/exp | 17420 | 22-Mar-22 |
| #10 | 'respiratory distress syndrome'/exp | 92791 | 22-Mar-22 |
| #9 | #1 OR #2 OR #3 OR #4 OR #5 OR #6 OR #7 OR #8 | 264578 | 22-Mar-22 |
| #8 | 'sars coronavirus 2':ti,ab,kw OR 'sars‐like coronavirus':ti,ab,kw OR 'severe acute respiratory syndrome coronavirus‐2':ti,ab,kw | 22616 | 22-Mar-22 |
| #7 | sars‐cov‐2:ti,ab,kw OR sars‐cov2:ti,ab,kw OR sarscov2:ti,ab,kw OR sarscov‐2:ti,ab,kw | 85064 | 22-Mar-22 |
| #6 | wuhan:ti,ab,kw AND (disease:ti,ab,kw OR virus:ti,ab,kw OR coronavir*:ti,ab,kw OR 'corona vir*':ti,ab,kw) | 7150 | 22-Mar-22 |
| #5 | (new:ti,ab,kw OR novel:ti,ab,kw) AND (coronavir*:ti,ab,kw OR 'corona vir*':ti,ab,kw) | 30078 | 22-Mar-22 |
| #4 | (2019:ti,ab,kw OR 19:ti,ab,kw) AND (ncov:ti,ab,kw OR 'novel cov':ti,ab,kw OR covid:ti,ab,kw OR coronavir*:ti,ab,kw OR 'corona vir*':ti,ab,kw) | 223982 | 22-Mar-22 |
| #3 | covid19:ti,ab,kw OR 2019ncov:ti,ab,kw OR ncov2019:ti,ab,kw OR 19ncov:ti,ab,kw OR ncov19:ti,ab,kw | 216611 | 22-Mar-22 |
| #2 | 'severe acute respiratory syndrome coronavirus 2'/exp | 59472 | 22-Mar-22 |
| #1 | 'coronavirus disease 2019'/exp | 197768 | 22-Mar-22 |
