# Supplementary material for: Efficacy and safety of human umbilical cord-derived mesenchymal stem cells for COVID-19 pneumonia: a meta-analysis of randomized controlled trials
Source: Stem Cell Res Ther. 2023 May 4;14:118. doi: 10.1186/s13287-023-03286-8 (PMC10159228; doi:10.1186/s13287-023-03286-8)
Supplement: Supplementary file 3 — Additional file 3. Table S3. Search phrases for Web of Science. [file 13287_2023_3286_MOESM3_ESM.docx]

**Table S3.** search phrases for Web of Science

**COVID-19**

OR COVID 19 OR SARS-CoV-2 Infection OR Infection, SARS-CoV-2 OR SARS CoV 2 Infection OR SARS-CoV-2 Infections OR 2019 Novel Coronavirus Disease OR 2019 Novel Coronavirus Infection OR 2019-nCoV Disease OR 2019 nCoV Disease OR 2019-nCoV Diseases OR Disease, 2019-nCoV OR COVID-19 Virus Infection OR COVID 19 Virus Infection OR COVID-19 Virus Infections OR Infection, COVID-19 Virus OR Virus Infection, COVID-19 OR Coronavirus Disease 2019 OR Disease 2019, Coronavirus OR Coronavirus Disease-19 OR Coronavirus Disease 19 OR Severe Acute Respiratory Syndrome Coronavirus 2 Infection OR SARS Coronavirus 2 Infection OR COVID-19 Virus Disease OR COVID 19 Virus Disease OR COVID-19 Virus Diseases OR Disease, COVID-19 Virus OR Virus Disease, COVID-19 OR 2019-nCoV Infection OR 2019 nCoV Infection OR 2019-nCoV Infections OR Infection, 2019-nCoV OR COVID19 OR COVID-19 Pandemic OR COVID 19 Pandemic OR Pandemic, COVID-19 OR COVID-19 Pandemics

**SARS-CoV-2** OR

SARS Coronavirus 2 OR

Coronavirus 2, SARS OR

Coronavirus Disease 2019 Virus OR

2019 Novel Coronavirus OR

2019 Novel Coronaviruses OR

Coronavirus, 2019 Novel OR

Novel Coronavirus, 2019 OR

Wuhan Seafood Market Pneumonia Virus OR

SARS-CoV-2 Virus OR

SARS CoV 2 Virus OR

SARS-CoV-2 Viruses OR

Virus, SARS-CoV-2 OR

2019-nCoV OR

COVID-19 Virus OR

COVID 19 Virus OR

COVID-19 Viruses OR

Virus, COVID-19 OR

Wuhan Coronavirus OR

Coronavirus, Wuhan OR

COVID19 Virus OR

COVID19 Viruses OR

Virus, COVID19 OR

Viruses, COVID19 OR

Severe Acute Respiratory Syndrome Coronavirus 2

**Acute Lung Injury OR**

Acute Lung Injuries **OR**

Lung Injuries, Acute **OR**

Lung Injury, Acute

**Respiratory Distress Syndrome OR**

Distress Syndrome, Respiratory **OR**

Distress Syndromes, Respiratory **OR**

Respiratory Distress Syndromes **OR**

Syndrome, Respiratory Distress **OR**

Shock Lung **OR**

Lung, Shock **OR**

Respiratory Distress Syndrome, Acute **OR**

Acute Respiratory Distress Syndrome **OR**

ARDS, Human **OR**

Human ARDS **OR**

Respiratory Distress Syndrome, Pediatric **OR**

Pediatric Respiratory Distress Syndrome **OR**

Respiratory Distress Syndrome, Adult **OR**

Adult Respiratory Distress Syndrome

**pneumonia OR**

Pneumonias **OR**

Lobar Pneumonia **OR**

Lobar Pneumonias **OR**

Pneumonias, Lobar **OR**

Pneumonia, Lobar **OR**

Experimental Lung Inflammation **OR**

Experimental Lung Inflammations **OR**

Inflammation, Experimental Lung **OR**

Lung Inflammation, Experimental **OR**

Lung Inflammations, Experimental **OR**

Pneumonitis **OR**

Pneumonitides **OR**

Pulmonary Inflammation **OR**

Inflammation, Pulmonary **OR**

Inflammations, Pulmonary **OR**

Pulmonary Inflammations **OR**

Lung Inflammation **OR**

Inflammation, Lung **OR**

Inflammations, Lung **OR**

Lung Inflammations

**Critical Illness OR**

Critical Illnesses **OR**

Illness, Critical **OR**

Illnesses, Critical **OR**

Critically Ill

**Mesenchymal Stem Cells OR**

Stem Cell, Mesenchymal OR

Mesenchymal Stem Cell OR

Stem Cells, Mesenchymal OR

Bone Marrow Mesenchymal Stem Cells OR

Bone Marrow Mesenchymal Stem Cell OR

Bone Marrow Stromal Cells OR

Bone Marrow Stromal Cell OR

Bone Marrow Stromal Cells, Multipotent OR

Multipotent Bone Marrow Stromal Cell OR

Multipotent Bone Marrow Stromal Cells OR

Adipose-Derived Mesenchymal Stem Cells OR

Adipose Derived Mesenchymal Stem Cells OR

Adipose-Derived Mesenchymal Stromal Cells OR

Adipose Derived Mesenchymal Stromal Cells OR

Mesenchymal Stem Cells, Adipose-Derived OR

Mesenchymal Stem Cells, Adipose Derived OR

Adipose-Derived Mesenchymal Stem Cell OR

Adipose Derived Mesenchymal Stem Cell OR

Adipose Tissue-Derived Mesenchymal Stem Cell OR

Adipose Tissue Derived Mesenchymal Stem Cell OR

Adipose Tissue-Derived Mesenchymal Stem Cells OR

Adipose Tissue Derived Mesenchymal Stem Cells OR

Adipose Tissue-Derived Mesenchymal Stromal Cells OR

Adipose Tissue Derived Mesenchymal Stromal Cells OR

Adipose Tissue-Derived Mesenchymal Stromal Cell OR

Adipose Tissue Derived Mesenchymal Stromal Cell OR

Mesenchymal Stromal Cells OR

Mesenchymal Stromal Cell OR

Stromal Cell, Mesenchymal OR

Stromal Cells, Mesenchymal OR

Multipotent Mesenchymal Stromal Cells OR

Multipotent Mesenchymal Stromal Cell OR

Mesenchymal Stromal Cells, Multipotent OR

Mesenchymal Progenitor Cell OR

Mesenchymal Progenitor Cells OR

Progenitor Cell, Mesenchymal OR

Progenitor Cells, Mesenchymal OR

Wharton Jelly Cells OR

Wharton's Jelly Cells OR

Wharton's Jelly Cell OR

Whartons Jelly Cells OR

Bone Marrow Stromal Stem Cells

TS=(randomised OR randomized OR randomisation OR randomisation OR placebo* OR (random* AND (allocat* OR assign*)) OR (blind* AND (single OR double OR treble OR triple)))
