# Supplementary material for: Efficacy and safety of human umbilical cord-derived mesenchymal stem cells for COVID-19 pneumonia: a meta-analysis of randomized controlled trials
Source: Stem Cell Res Ther. 2023 May 4;14:118. doi: 10.1186/s13287-023-03286-8 (PMC10159228; doi:10.1186/s13287-023-03286-8)
Supplement: Supplementary file 4 — Additional file 4. Table S4. Search phrases for the Cochrane Library. [file 13287_2023_3286_MOESM4_ESM.docx]

**Table S4.** search phrases for the Cochrane Library

Search Name: MSC AND COVID-19 ARDS ALI

Last Saved: 22/03/2022 18:35:17

Comment:

ID Search

#1 MeSH descriptor: [COVID-19] explode all trees

#2 MeSH descriptor: [SARS-CoV-2] explode all trees

#3 (COVID19 OR 2019nCoV OR nCoV2019 OR 19nCoV OR ncov19):ti,ab,kw (Word variations have been searched)

#4 ((2019 OR 19) AND (nCoV OR novel CoV OR COVID OR coronavir* OR ‘corona vir*’)):ti,ab,kw (Word variations have been searched)

#5 ((New OR novel) AND (coronavir* OR ‘corona vir* ‘)):ti,ab,kw (Word variations have been searched)

#6 (wuhan AND (disease or virus or coronavir* OR ‘corona vir*‘)):ti,ab,kw (Word variations have been searched)

#7 (("SARS‐CoV‐2" or "SARS‐CoV2" or SARSCoV2 or "SARSCoV‐2")):ti,ab,kw (Word variations have been searched)

#8 (("SARS coronavirus 2" or "SARS‐like coronavirus" or "Severe Acute Respiratory Syndrome Coronavirus‐2")):ti,ab,kw (Word variations have been searched)

#9 #1 OR #2 OR #3 OR #4 OR #5 OR #6 OR #7 OR #8

#10 MeSH descriptor: [Acute Lung Injury] explode all trees

#11 MeSH descriptor: [Respiratory Distress Syndrome] explode all trees

#12 MeSH descriptor: [Pneumonia] explode all trees

#13 MeSH descriptor: [Critical Illness] explode all trees

#14 ((respirat* or ventilat*) AND (distress* or depress* or failure* or insufficien* or paraly*)):ti,ab,kw (Word variations have been searched)

#15 ((pulmonary* or lung* or alveol*) AND (collapse* or injur* or failure* or damage* or shock)):ti,ab,kw (Word variations have been searched)

#16 ((acute or serious or severe) AND (hypox* or respirat*)):ti,ab,kw (Word variations have been searched)

#17 (acute AND (lung injur* or distress syndrome*)):ti,ab,kw (Word variations have been searched)

#18 (Pneumoni*):ti,ab,kw (Word variations have been searched)

#19 ((Lung* OR pulmonary*) AND (Inflammat* OR infect*)):ti,ab,kw (Word variations have been searched)

#20 (Moderate* OR common* OR severe* OR critical*):ti,ab,kw (Word variations have been searched)

#21 (ALI OR ARDS):ti,ab,kw (Word variations have been searched)

#22 #10 OR #11 OR #12 OR #13 OR #14 OR #15 OR #16 OR #17 OR #18 OR #19 OR #20 OR #21

#23 #9 AND #22

#24 MeSH descriptor: [Mesenchymal Stem Cells] explode all trees

#25 (“Mesenchymal stem cell*”):ti,ab,kw (Word variations have been searched)

#26 (“wharton* Jelly cell*”):ti,ab,kw (Word variations have been searched)

#27 ((mesenchymal OR adipose OR marrow OR placenta* OR “umbilical cord” OR “Dental pulp” OR “Wharton’s Jelly” ) AND (stem OR stroma* OR Progenitor*) AND cell*):ti,ab,kw (Word variations have been searched)

#28 ((multipotent OR multi-potent) AND (stem OR stroma* ) AND cell*):ti,ab,kw (Word variations have been searched)

#29 (MSC* OR AD-MSC* OR AT-MSC* OR ADSC* OR UC-MSC* OR WJ-MSC* OR BM-MSC* OR DP-MSC* OR PL-MSC* OR DSC* OR hMSC*):ti,ab,kw (Word variations have been searched)

#30 #24 OR #25 OR #26 OR #27 OR #28 OR #29

#31 #23 AND #30
