# Supplementary material for: Efficacy and safety of human umbilical cord-derived mesenchymal stem cells for COVID-19 pneumonia: a meta-analysis of randomized controlled trials
Source: Stem Cell Res Ther. 2023 May 4;14:118. doi: 10.1186/s13287-023-03286-8 (PMC10159228; doi:10.1186/s13287-023-03286-8)
Supplement: Supplementary file 5 — Additional file 5. Table S5. Summary of included patients. [file 13287_2023_3286_MOESM5_ESM.docx]

**Table S5.** Summary of included patients

| **Year** | | **Author** | **Comorbidities** | | **Admission symptoms** | | **Time from symptom onset to starting study treatment (days)** | | **baseline pulmonary function** | | **Baseline general MSC; Ctrl** | | **baseline laboratory examinations** | | **baseline therapy** | | **Covid-19 severity** | |
| --- | --- | --- | --- | --- | --- | --- | --- | --- | --- | --- | --- | --- | --- | --- | --- | --- | --- | --- |
|  |  | | **MSC** | **Ctrl** | **MSC** | **Ctrl** | **MSC** | **Ctrl** | **MSC** | **Ctrl** | **MSC** | **Ctrl** | **MSC** | **Ctrl** | **MSC** | **Ctrl** | **MSC** | **Ctrl** |
| 2021 | Zhu et al | | CHD (3) DM (4) Cerebrovasc (3) Hypertension (12) Chron Respir Dis (1) History of LD and KD (2) | CHD (3) DM (4) Cerebrovasc (2) Hypertension (11) Chron Respir Dis (0) History of LD and KD (3) | Cough (22) Fever (16) Anhelation (17) Chest distress (11) Fatigue (21) Muscular soreness (9) Poor appetite (3) Diarrhea (3) Dizziness (2) Nausea and vomiting (3) | Cough (21) Fever (20) Anhelation (16) Chest distress (14) Fatigue (19) Muscular soreness (5) Poor appetite (5) Diarrhea (3) Dizziness (5) Nausea and vomiting (3) | 13 (9.5, 15.5) | 11 (8, 14.5) | RR 20 (18, 21) | RR 20 (19, 20) | TEMP 36.7 (36.5, 38.0)  PR 78 (75.0, 86)  SBP 130 (118, 136)  DBP 79 (75, 81) | TEMP 36.6 (36.4, 36.8)  PR 80 (77, 90)  SBP 128 (119, 137)  DBP 74 (70, 80) | CRP 11.1 (9.16, 15.2) PCT 0.10 (0.04, 0.14) WBC 6.31 (4.20, 7.37) NEUT 5.66 (3.40, 7.48) LYM 0.64 (0.42, 1.12) MON (/uL) 0.25 (0.19, 0.48) | CRP 55.2 (32.0, 110.2) PCT 0.09 (0.04, 0.17) WBC 6.75 (4.92, 8.64) NEUT 4.34 (2.91, 5.95) LYM 0.93 (0.54, 1.24) MON (/uL) 0.30 (0.20, 0.44) | OT (27) NIMV (3) IMVn (0) Corticosteroids (20)  Antibiotic (18) AV (13) | OT (24) NIMV (2) IMVn (0) Corticosteroids (19) Antibiotic (19) AV (17) | Common/mild (15)  Severe (11) Critical (3) | Common/mild (16)  Severe (10) Critical (3) |
| 2020 | Shu et al | | DM (3) Hypertension (3) | DM (5) Hypertension (6) | Fever (10) Cough (8) | Fever (26)  Cough (19) | 11.50 (6.00, 20.00) | 14.00 (10.00, 18.00) | CT score 18.50 (16.25, 20.75)  OI 197.08 ± 33.42 RR > 24/min (11) | Ctrl:  CT score 16.00 (15.00, 20.00)  OI 172.01 ± 27.85 RR > 24/min (20) | NEWS2 9.0 (8.00, 10.75) | NEWS2 8.00 (7.00, 10.00) | WBC 7.37 (5.06, 11.16) LYM 0.77 (0.43, 1.72) MON 0.41 (0.26, 0.65) LDH 285.5 (220.0, 392.0)  CRP 68.4 ± 25.8 IL-6 62.1 ± 34.4 | WBC 6.88 (5.06, 8.71) LYM 0.82 (0.59, 1.11) MON 0.62 (0.33, 0.0.91) LDH 331.0 (237.5, 441.0)  CRP 32.6 ± 26.2 IL-6 37.6 ± 40.8 | OT (7) HFNC or NIMV (4) ECMO, IMV (0) | OT (21) HFNC or NIMV (6) ECMO, IMV (0) | All severe | All severe |
| 2021 | Shi et al | | Hypertension (17) DM (12) CB (2) COPD (2) | Hypertension (10) DM (5) CB (3) COPD (0) | NR | NR | 45.00 (39.00, 51.00) | 47.00 (41.00, 53.00) | NR | NR | NR | NR | WBC 5.70 (5.00, 6.60)  LYM 1.39 (1.19, 1.80) CD4+ T cells 641.00 (482.00, 760.00) CD8+ T cells 371.00 (275.00, 520.00) B cells 148.50 (99.60, 251.00) NK cells 233.50 (151.00, 393.00)  NEUT 3.48 (2.91, 4.32)  IL-6 7.86 (5.63, 9.84)  CRP 1.95 (0.84, 3.53) | WBC 5.80 (5.00, 6.80)  LYM 1.47 (1.24, 1.84) CD4+ T cells 734.00 (502.00, 1031.00) CD8 T cells 401.00 (307.00, 593.00) B cells 148.50 (94.70, 248.00)  NK cells 197.50 (136.00, 309.00)  NEUT 3.83 (2.85, 4.48)  IL-6 8.76 (6.54, 11.77)  CRP 1.38 (0.68, 2.26) | AV (32) Antibiotics (27) Corticosteroids (13) OT (50) NIMV or HFOT 1 (1.54%) | AV (20) Antibiotics (12) Corticosteroids (9) OT (25) NIMV or HFOT 0 (0.00%) | All severe | All severe |
| 2022 | Monsel et al | | COPD (0) Smoking (0) CHF (0) AF (0) Hypertension (2) CAD (11) Stroke (2) Immunodeficiency (0) Active neoplasia (0) Obesity (7) | COPD (1) Smoking (0) CHF (0) AF (0) Hypertension (10) CAD (2) Stroke (1) Immunodeficiency (0) Active neoplasia (0) Obesity (6) | NR | NR | NR | NR | VS(NIV and/or HFNO) (10) IMV (11) VT 6.2 (0.7, n = 11)  PAP 21.8 (4.2, n = 10)  PEEP 10.8 (2.9, n = 11)  Driving pressure 11.3 (4.3, n = 10)  Compliance 45.2 (27.8, n = 10)  SpO2 94.6 (3.4) P/F 156.2 (68.2) LIS 3.0 (0.7) PaCO2 40 (8.5) pH 7.41 (0.1) | VS(NIV and/or HFNO) (4) IMV (20) VT 6.3 (0.8, n = 20)  PAP 24.8 (5.1, n = 17)  PEEP 11.2 (3.2, n = 20)  Driving pressure 13.2 (3.9, n = 17)  Compliance 35.2 (14.9, n = 17)  SpO2 96.0 (3.0, n = 23)  P/F 171.2 (72.9) LIS 2.8 (0.5) PaCO2 43.2 (9.8) pH 7.37 (0.1) | SOFA 5.5 (2.7) MAP 91.3 (18.3) | SOFA 5.9 (2.7)  MAP 81.5 (16.9) | NR | NR | Chronic corticosteroid intake (0) IMD (2) VS(NIV and/or HFNO) (10) IMV (11) Vasopressors (5) | Chronic corticosteroid intake (0) IMD (0) VS(NIV and/or HFNO) (4) IMV (20) On vasopressors (14) | All severe | All severe |
| 2021 | Lanzoni et al | | MSC DM (5) Hypertension (7) Obesity (11)  Cancer (0) HD 1 (8.3)  Smoker 0 (0) | Ctrl DM (6) Hypertension (9) Obesity (5) Cancer (1) HD 3 (25)  Smoker 2 (16.7) | NR | NR | NR | NR | P/F 124 (68, 164) | P/F 108.5 (68.5, 165.5) | NR | NR | IL-6 32 (6.96，76.52） IFN-γ 447.684 (319.039,622.642) | IL-6 126.61 (48.7，200.35) IFN-γ 509.434 (252.144, 782.161) | AV (9) Corticosteroids (10) IMD (1) | AV (7) Corticosteroids 9 (75)  IMD (4) | All severe | All severe |
| 2021 | Dilogo et al | | DM mellitus 8 Hypertension 6 CKD 2 CAD 2 CCF 1 Tuberculosis 1 Others^a^ 10 | Ctrl DM mellitus 12 Hypertension 10 CKD 5 CAD 3 CCF 1 Tuberculosis 1 Others^a^ 6 | NR | NR | NR | NR | NR | NR | NR | NR | NR | NR | NR | NR | All critical | All critical |
| 2021 | Adas et al | | NR | NR | NR | NR | NR | NR | NR | NR | NR | NR | CRP 159.5 ± 30.1 PCT 1.7 ± 2  IFN-γ 80.2 ± 14.7 IL-6 101 ± 28.2  IL-17A 82.2 ± 15.2 IL-2 131.2 ± 29.1  IL-12 11.4 ± 4.2  IL-10 26.5 ± 21.4  IL-13 630 ± 150.2  IL-1ra 3134.2 ± 1522.6 | CRP 165 ± 63  PCT 1.5 ± 1.9  IFN-γ 74.2 ± 8.1  IL-6 97 ± 38.3  IL-17A 86 ± 18  IL-2 127.7 ± 27  IL-12 10.3 ± 3.8  IL-10 20.5 ± 15.8  IL-13 745 ± 231.5  IL-1ra 3363.4 ± 2139.3 | NR | NR | All critical | All critical |

CHD: coronary heart disease; DM, diabetes mellitus; Cerebrovasc, cerebrovascular disease; Chron Respir Dis, chronic respiratory disease; LD, liver disease; KD, kidney disease; CB, chronic bronchitis; COPD, chronic obstructive pulmonary disease; CHF, chronic heart failure; AF, atrial fibrillation; CAD, coronary artery/arterial disease; HD, Heart disease; CKD, chronic kidney disease; CCF, congestive cardiac failure; OI, oxygenation index; RR, respiratory rate, times/min; VS, ventilatory support; IMV, invasive mechanical ventilation; VT, Tidal volume, mL/kg PBW; PAP, Plateau airway pressure, cmH2O; PEEP, Positive End Expiratory Pressure; SpO2, percutaneous arterial oxygen saturation, %; P/F, PaO2/FiO2, mmHg; LIS, Lung injury score; PaCO2, the partial pressure of carbon dioxide, mm Hg; Compliance, mL/cm H2O; TEMP, temperature, °C; PR, pulse rate, times/min; SBP, systolic blood pressure, mmHg; DBP, diastolic blood pressure, mmHg; NEWS2, the national early warning score; SOFA, sepsis‐related organ‐failure assessment score; MAP, mean arterial pressure, mm Hg; CRP, c-reactive protein, mg/L; PCT, procalcitonin, ng/mL; WBC, white blood cell, × 10^9^/L; NEUT, neutrophils, × 10^9^/L;LYM, lymphocyte, × 10^9^/L; MON, monocytes, × 10^9^/L; LDH, lactate dehydrogenase, U/L; IL-6, Interleukin-6, pg/ml; IL-17A, Interleukin-17A, pg/m; IL-2, Interleukin-2, pg/ml;IL-12, Interleukin-12, pg/ml; IL-10, Interleukin-10, pg/ml; IL-13, Interleukin-13, pg/ml; IL-1ra, Interleukin-1ra, pg/ml; OT, Oxygen therapy; T cells, T lymphocyte, /μl; B cells, B lymphocyte, /μl; NK cells, natural killer cells, /μl; IFN-γ, Interferon-gamma, pg/ml; NIMV, Non-Invasive Mechanical Ventilation; AV, Anti-virus therapy; HFOT, high flow oxygen devices, ECMO, Extracorporeal Membrane Oxygenation; HFNC, Humidified High Flow Nasal Cannula; HFNO, High-flow nasal oxygen; IMD, immunomodulatory drugs; NR, not reported

Other^a^ comorbidities include gastric perforation, pleural effusion, multiple rib fractures, obesity, hypercoagulation, and lung contusion in the Recovered group and icterus, stroke infarction, Disseminated Intravascular Coagulation (DIC), atrial fibrillation, obesity, acute kidney injury, myocardial infarction, and hypertensive heart disease in the Died group.
